# Supplementary material for: Characterization of viscoelastic behavior of basalt fiber asphalt mixtures based on discrete and continuous spectrum models
Source: PLoS One. 2024 Feb 1;19(2):e0296087. doi: 10.1371/journal.pone.0296087 (PMC10833572; doi:10.1371/journal.pone.0296087)
Supplement: Attachment — Submitted filename: PONE-D-23-33461...with comments.pdf [file pone.0296087.s001.pdf]

PLOS ONE

Characterization of viscoelastic behavior of basalt fiber asphalt mixtures based on discrete and continuous spectrum models

--Manuscript Draft--

**Manuscript Number:**	PONE-D-23-33461
**Article Type:**	Research Article
**Full Title:**	Characterization of viscoelastic behavior of basalt fiber asphalt mixtures based on discrete and continuous spectrum models
**Short Title:**	comprehensively characterizing the viscoelastic behavior of basalt fiber asphalt mixtures
**Corresponding Author:**	xioagang kang<br>North China University of Water Resources and Electric Power<br>郑州, CHINA
**Keywords:**	Basalt fiber asphalt mixtures; Master curves of viscoelastic parameters; Discrete spectrum; Continuous spectrum
**Abstract:**	In order to analyze the differences between the master curves of relaxation modulus and creep compliance obtained from discrete and continuous spectrum models, and to comprehensively evaluate the effect of basalt fiber content on the viscoelastic behavior of asphalt mixtures, complex modulus tests were conducted for asphalt mixtures with fiber content of 0%, 0.1%, 0.2% and 0.3%, respectively. Consequently, the master curves of Viscoelastic Parameters of asphalt mixtures in the frequency domain were constructed according to the generalized Sigmoidal model(GSM) and the approximate Kramers-Kronig (K-K) relationship. Then, transformation of master curves using discrete and continuous spectrum models to obtain the models of and containing all viscoelastic information. Also, the accuracy of the models of and was evaluated. The results show that two types of spectrum can be obtained highly accurate models of and with little difference in accuracy. The addition of basalt fibers can enhance the strength, stress relaxation and deformation resistance of asphalt mixtures. In addition, the basalt fiber achieves the purpose of enhancing its low-temperature crack resistance by changing the internal structure of the asphalt mixtures. And, when the content of basalt fiber in the linear viscoelasticity range is 0.2%, the asphalt mixtures has the best low-temperature stress relaxation ability and high-temperature deformation resistance.
**Order of Authors:**	QI huang<br>xioagang kang<br>zhengji zhang<br>pengfei chen<br>zhuohang zhang<br>ErHu Yan
**Additional Information:**	
**Question**	**Response**
**Financial Disclosure**<br><br>Enter a financial disclosure statement that describes the sources of funding for the work included in this submission. Review the submission guidelines for detailed requirements. View published research articles from *PLOS ONE* for specific examples.	The author(s) received no specific funding for this work.

Powered by Editorial Manager® and ProduXion Manager® from Aries Systems Corporation

This statement is required for submission and **will appear in the published article** if the submission is accepted. Please make sure it is accurate.<br><br>**Funded studies**<br>Enter a statement with the following details:<br>• Initials of the authors who received each award<br>• Grant numbers awarded to each author<br>• The full name of each funder<br>• URL of each funder website<br>• Did the sponsors or funders play any role in the study design, data collection and analysis, decision to publish, or preparation of the manuscript?<br><br>Did you receive funding for this work?	
**Competing Interests**<br><br>Use the instructions below to enter a competing interest statement for this submission. On behalf of all authors, disclose any competing interests that could be perceived to bias this work—acknowledging all financial support and any other relevant financial or non-financial competing interests.<br><br>This statement is required for submission and **will appear in the published article** if the submission is accepted. Please make sure it is accurate and that any funding sources listed in your Funding Information later in the submission form are also declared in your Financial Disclosure statement.<br><br>View published research articles from *PLOS ONE* for specific examples.	The authors have declared that no competing interests exist

Powered by Editorial Manager® and ProduXion Manager® from Aries Systems Corporation

**NO authors have competing interests**<br><br>Enter: *The authors have declared that no competing interests exist.*<br><br>**Authors with competing interests**<br><br>Enter competing interest details beginning with this statement:<br><br>*I have read the journal's policy and the authors of this manuscript have the following competing interests: [insert competing interests here]*<br><br>* typeset	
**Ethics Statement**<br><br>Enter an ethics statement for this submission. This statement is required if the study involved:<br><br>• Human participants<br>• Human specimens or tissue<br>• Vertebrate animals or cephalopods<br>• Vertebrate embryos or tissues<br>• Field research<br><br>Write "N/A" if the submission does not require an ethics statement.<br><br>General guidance is provided below. Consult the submission guidelines for detailed instructions. **Make sure that all information entered here is included in the Methods section of the manuscript.**	N/A

Powered by Editorial Manager® and ProduXion Manager® from Aries Systems Corporation

**Format for specific study types**<br><br>**Human Subject Research (involving human participants and/or tissue)**<br>• Give the name of the institutional review board or ethics committee that approved the study<br>• Include the approval number and/or a statement indicating approval of this research<br>• Indicate the form of consent obtained (written/oral) or the reason that consent was not obtained (e.g. the data were analyzed anonymously)<br><br>**Animal Research (involving vertebrate animals, embryos or tissues)**<br>• Provide the name of the Institutional Animal Care and Use Committee (IACUC) or other relevant ethics board that reviewed the study protocol, and indicate whether they approved this research or granted a formal waiver of ethical approval<br>• Include an approval number if one was obtained<br>• If the study involved *non-human primates*, add *additional details* about animal welfare and steps taken to ameliorate suffering<br>• If anesthesia, euthanasia, or any kind of animal sacrifice is part of the study, include briefly which substances and/or methods were applied<br><br>**Field Research**<br><br>Include the following details if this study involves the collection of plant, animal, or other materials from a natural setting:<br>• Field permit number<br>• Name of the institution or relevant body that granted permission	
**Data Availability**<br><br>Authors are required to make all data underlying the findings described fully available, without restriction, and from the time of publication. PLOS allows rare exceptions to address legal and ethical concerns. See the PLOS Data Policy and FAQ for detailed information.	Yes - all data are fully available without restriction

A Data Availability Statement describing where the data can be found is required at submission. Your answers to this question constitute the Data Availability Statement and **will be published in the article**, if accepted.<br><br>**Important:** Stating 'data available on request from the author' is not sufficient. If your data are only available upon request, select 'No' for the first question and explain your exceptional situation in the text box.<br><br>Do the authors confirm that all data underlying the findings described in their manuscript are fully available without restriction?	
**Describe where the data may be found in full sentences. If you are copying our sample text, replace any instances of XXX with the appropriate details.**<br><br>• If the data are **held or will be held in a public repository**, include URLs, accession numbers or DOIs. If this information will only be available after acceptance, indicate this by ticking the box below. For example: *All XXX files are available from the XXX database (accession number(s) XXX, XXX.)*.<br>• If the data are all contained **within the manuscript and/or Supporting Information files**, enter the following: *All relevant data are within the manuscript and its Supporting Information files.*<br>• If neither of these applies but you are able to provide **details of access elsewhere**, with or without limitations, please do so. For example:<br><br>*Data cannot be shared publicly because of [XXX]. Data are available from the XXX Institutional Data Access / Ethics Committee (contact via XXX) for researchers who meet the criteria for access to confidential data.*<br><br>*The data underlying the results presented in the study are available from (include the name of the third party*	All relevant data are within the manuscript and its Supporting Information files

and contact information or URL).<br>• This text is appropriate if the data are owned by a third party and authors do not have permission to share the data.<br><br>* typeset	
Additional data availability information:	

Manuscript Click here to access/download;Manuscript;article.docx

Characterization of viscoelastic behavior of basalt fiber asphalt mixtures based on discrete and continuous spectrum models

Qi Huang[1], Xiaogang Kang[2], Zhengji Zhang[3], Pengfei Chen[4], Zhuohang Zang[5] Er-hu Yan[6]

[1]Professor, School of Civil Engineering and Communication, North China University of Water Resources and Electric Power, Zhengzhou 450045, P. R. China(corresponding author). Email: hq66138@163.com.

[2] Graduate Research Assistant, School of Civil Engineering and Communication, North China University of Water Resources and Electric Power, Zhengzhou 450045, China. Email: 13781229603@163.com.

[3] Graduate Research Assistant, School of Civil Engineering and Communication, North China University of Water Resources and Electric Power, Zhengzhou 450045, China. Email: 19603778185@163.com.

[4] Graduate Research Assistant, School of Civil Engineering and Communication, North China University of Water Resources and Electric Power, Zhengzhou 450045, China. Email: cpf1145266694@163.com.

[5] Graduate Research Assistant, School of Civil Engineering and Communication, North China University of Water Resources and Electric Power, Zhengzhou 450045, China. Email: m15638253165@163.com.

[6] Researcher ,Research Institute of Highway, Ministry of Transport, Beijing 100088, China. Email: eh.yan@rioh.cn.

Abstract: In order to analyze the differences between the master curves of relaxation modulus $E(t)$ and creep compliance $J(t)$ obtained from discrete and continuous spectrum models, and to comprehensively evaluate the effect of basalt fiber content on the viscoelastic behavior of asphalt mixtures, complex modulus tests were conducted for asphalt mixtures with fiber content of 0%, 0.1%, 0.2% and 0.3%, respectively. Consequently, the master curves of Viscoelastic Parameters of asphalt mixtures in the frequency domain were constructed according to the generalized Sigmoidal model(GSM) and the approximate Kramers-Kronig (K-K) relationship. Then, transformation of master curves using discrete and continuous spectrum models to obtain the models of $E(t)$ and $J(t)$ containing all viscoelastic information. Also, the accuracy of the models of $E(t)$ and $J(t)$ was evaluated. The results show that two types of spectrum can be obtained highly accurate models of $E(t)$ and $J(t)$ with little difference in accuracy. The addition of basalt fibers can enhance the strength, stress relaxation and deformation resistance of asphalt mixtures. In addition, the basalt fiber achieves the purpose of enhancing its low-temperature crack resistance by changing the internal structure of the asphalt mixtures. And, when the content of basalt fiber in the linear viscoelasticity range is 0.2%, the asphalt mixtures has the best low-temperature stress relaxation ability and high-temperature deformation resistance.

Key words: Basalt fiber asphalt mixtures; Master curves of viscoelastic parameters; Discrete spectrum; Continuous spectrum

1 Introduction

The viscoelastic behavior of asphalt mixture directly affects the road performance of asphalt pavement[1]. Dynamic modulus $|E^*(\omega)|$, storage modulus $E'(\omega)$, loss modulus $E''(\omega)$, relaxation modulus $E(t)$, and creep compliance $J(t)$ are commonly used to describe the viscoelastic behavior of asphalt mixtures[2]. Different viscoelastic parameters characterize the different viscoelastic response. Among them, $|E^*(\omega)|$, $E'(\omega)$ and $E''(\omega)$ are all functions with respect to frequency and

describe the strength properties, purely elastic behavior and purely viscous behavior of the asphalt mixtures, respectively[3]. $E(t)$ and $J(t)$ are all functions of time and describe the stress relaxation and deformation capacity of the asphalt mixture, respectively[4].

Meanwhile, $|E^*(\omega)|$ is an important parameter for asphalt pavement design, and $E(t)$ and $J(t)$ can be used to predict the temperature stress and deformation of asphalt pavements[5]. However, the cost of obtaining accurate $E(t)$ and $J(t)$ is significant due to factors such as test instrumentation, test time and test operability. In contrast, complex modulus tests are simpler to operate and the tests are more easily controlled[6]. According to the theory of linear viscoelasticity, these response functions are interrelated, and the conversion of viscoelastic parameters to each other can be realized through mathematical theories[7]. Therefore, it is practical to constant the master curves of $E(t)$ and $J(t)$ from the linear viscoelasticity theory and the complex modulus test results[8] .

The Boltzmann superposition principle provides a theoretical basis for the conversion between the models of the viscoelastic parameters[9]. The models of $E(t)$ and $J(t)$ are usually determined tests using discrete and continuous models[10][11]. The most commonly used discrete models of $E(t)$ and $J(t)$ is the Prony series model. Using this model to obtain the model of $E(t)$ and $J(t)$ mainly includes four steps. First, the master curves of storage compliance $E'(\omega)$ and storage compliance $J'(\omega)$ are constructed with the purpose of pre-smoothing the experimental data. Second, the Prony series model of $E'(\omega)$ is derived from the generalized Maxwell model(GMM), and the Prony series model of $J'(\omega)$ is derived from the generalized Kelvin model(GKM)[12]. Third, the coefficients in the Prony series were determined by fitting the models of $E'(\omega)$ and $J'(\omega)$. Finally, the discrete model of $J(t)$ is determined according to the transformation relationship between $E'(\omega)$ and $E(t)$; and ,the discrete model of $J(t)$ is determined according to the transformation relationship between $J'(\omega)$ and $J(t)$.

Correspondingly, five steps are required to determine $E(t)$ and $J(t)$ using the continuous spectrum models[13][15]. First, the models of $E'(\omega)$ and $J'(\omega)$ are selected and fitted. Second, an expression for the continuous relaxation spectrum $H(\rho)$ with respect to the model of $E'(\omega)$ is derived from the relationship between $E'(\omega)$ with $H(\rho)$. And the expression of the continuous retardation spectrum $L(\tau)$ with respect to the model of $J'(\omega)$ is derived from the relationship of $J'(\omega)$ with $L(\tau)$. Third, the continuous relaxation and retardation spectrum models are determined by substituting the models of $E'(\omega)$ and $J'(\omega)$ into this expressions, respectively. Fourth, the equilibrium modulus is determined from the model of $E'(\omega)$ and the expression of $E'(\omega)$ with

respect to the continuous relaxation spectrum. And the glassy compliance is determined from the model of $J'(\omega)$ and the expression of $J'(\omega)$ with respect to the continuous retardation spectrum. Finally, the numerical models of $E(t)$ and $J(t)$ are established by using the numerical integral formula.

There are two main drawbacks of using the above discrete and continuous spectrum models for the master curve conversion of viscoelastic parameters. First, the conversion process ignores the information provided by loss modulus and loss compliance, and the constructed the master curves of $E(t)$ and $J(t)$ do not contain all the viscoelastic information. Secondly, fitting the models of $E'(\omega)$ and $J'(\omega)$ separately increases the number of fits and reduces the accuracy of the converted master curves.

Basalt fiber, as a new road material, has received a lot of attention in recent years. Previous studies on the viscoelastic behavior of basalt fiber asphalt mixtures have more often used dynamic modulus and phase angle to evaluate their viscoelastic behavior, and rarely studied their viscoelastic properties comprehensively[16][17]. Moreover, many studies characterizing the viscoelasticity of basalt fiber asphalt mixtures only plot the dynamic modulus master curve and ignore the phase angle master curve; or choose two unrelated models to plot the dynamic modulus and phase angle master curves separately. The model functions thus obtained do not satisfy the K-K relationship or do not conform to the linear viscoelasticity theory. The research methods for the relaxation and deformation properties of asphalt mixtures are also limited to static tests that differ significantly from the actual road stresses in service.

In view of the above problems, to evaluate the difference of the master curve obtained by the transformation of discrete and continuous models, and to achieve the purpose of comprehensively characterizing the viscoelastic behavior of basalt fiber asphalt mixtures. In this study, the approximate Kramers-Kronig(K-K)relationship and the generalized Sigmoidal model(GSM) are applied to construct the master curves of dynamic modulus, phase angle, storage modulus and loss modulus. Then, the master curves of $E(t)$ containing all viscoelastic information are constructed from the master curves of $E'(\omega)$ and $E''(\omega)$ using discrete and continuous spectrum models, respectively. Next, the master curves of $J(t)$ is derived from the integral relationship between the $E(t)$ and $J(t)$ in the time domain. Finally, the accuracy of the master curves obtained from the discrete and continuous spectrum models is evaluated.

2 Materials and test methods

2.1 Materials

The type of asphalt mixtures used in this study was AC-13 dense grading asphalt mixtures, as shown in Figure 1. The asphalt used No. 70 base asphalt produced in Tianjin. The coarse and fine aggregates and mineral powder come from limestone produced in Tongzhou. In this study, the fiber content was selected as 0%, 0.1%, 0.2% and 0.3% (the fiber content accounted for the mass fraction

of the asphalt mixture), and for the convenience of the study, they were numbered as BF-0, BF-1, BF-2 and BF-3 ,respectively. The basalt fiber asphalt mixture is prepared by dry mixing method to ensure that the basalt fiber is fully dispersed in the asphalt mixtures.

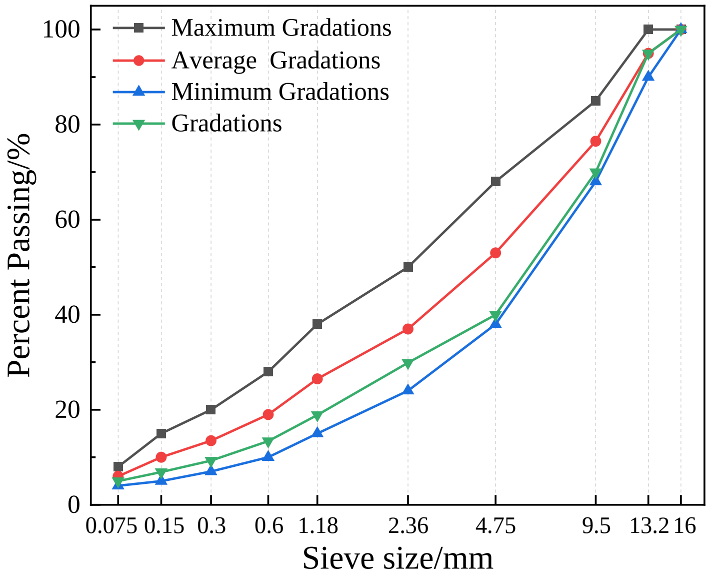

Fig.1 Basalt fiber asphalt mixtures gradation

2.2 Complex modulus tests

According to the AASHTO TP 79-12, the complex modulus test was carried out using the asphalt mixture performance tester (AMPT) produced by the Italian Matester Company[18]. The test temperature is -10°C, 20°C and 35°C. The dynamic modulus and phase angle of six loading frequencies of 25Hz, 10Hz, 5Hz, 1Hz, 0.5Hz, and 0.1Hz were measured at each temperature, and the loading sequence was sequentially loaded from high frequency to low frequency.

3 Theoretical background

3.1 Complex modulus test parameters

The complex modulus E^*, dynamic modulus $\left|E^*\right|$, storage modulus E', loss modulus E', and phase angle φ have the relationships shown in equations (1)-(4).

$$E^* = E' + iE'' \quad (1)$$

$$|E^*| = \sqrt{(E')^2 + (E')^2} \quad (2)$$

$$E' = |E^*| \cos\varphi \quad (3)$$

$$E'' = |E^*| \sin\varphi \quad (4)$$

3.2 The Time-Temperature Superposition Principle (TTSP)

The viscoelasticity of asphalt mixture has a strong dependence on time and temperature, and its viscoelastic behavior shows the equivalence of time and temperature, which is called TTSP[19]. When TTSP is applied, the test curves at different temperatures can be moved to the target temperature curve, and the moving distance is called the shift factor $\alpha(t)$. In this paper, when

constructing the master curves the Williams-Landel-Ferr (WFL) equation shown in Eq.(5) is chosen to calculate , b[illegible]use its parameters have clear physical meaning and the calculation results are more accurate[20].

$$\log \alpha_T = \frac{-C_1(T-T_r)}{C_2+(T-T_r)} \tag{5}$$

Where C_1, C_2 is the material constant; T is the test temperature; and T_r is the reference temperature.

3.3 Construction of master curves in frequency domain

3.3.1 Construction of the master curves of $\left|E^*(\omega)\right|$ and $\varphi(\omega)$

In order to ensure that the constructed the master curves of $\left|E^*(\omega)\right|$ and $\varphi(\omega)$ both conform to linear viscoelasticity and allow the master curves to be unsymmetrical about the viewpoint, the (GSM)and the approximate K-K relationship are used, as shown in Eq (6)-(8)[1][21].

$$\varphi(\omega) \approx \frac{\pi}{2} \frac{\mathrm{d} \ln |E^*(\omega)|}{\mathrm{d} \ln \omega} \tag{6}$$

$$\log |E^*(f_r)| = \delta + \frac{\alpha}{\left(1+\lambda e^{\beta+\gamma(\log f_r)}\right)^{\frac{1}{\lambda}}} \tag{7}$$

$$\log \varphi(f_r) = \log\left(-\frac{\pi}{2}\alpha\gamma\right) + \left[\beta e^{\beta+\gamma(\log f_r)}\right]\log e - \left(1+\frac{1}{\lambda}\right)\log\left[1+\lambda e^{\beta+\gamma(\log f_r)}\right] \tag{8}$$

where f_r is the reduction frequency, $\log f_r = \log f + \log \alpha_T$; δ is the maximum value of the $\left|E^*(\omega)\right|$, α is the difference between the maximum and minimum values of $\left|E^*(\omega)\right|$; β 、 γ and λ are the shape coefficients.

Selecting 20°C as the reference temperature, the model parameters of $\left|E^*(\omega)\right|$ and $\varphi(\omega)$ are simultaneously solved by minimizing the parameter error. The error function is

$$error = \frac{1}{n}\sqrt{\sum_{i=1}^{n}\left(\frac{\log\left|E^*\right|_{m,i} - \log\left|E^*\right|_{p,i}}{\log\left|E^*\right|_{m,i}}\right)^2} + \frac{1}{n}\sqrt{\sum_{i=1}^{n}\left(\frac{\log\varphi_{m,i} - \log\varphi_{p,i}}{\log\varphi_{m,i}}\right)^2} \tag{9}$$

where n is the total data points of the test, $n=18$; $\log\left|E^*\right|_{m,i}$ is the logarithm of the experimental value of $\left|E^*(\omega)\right|$; $\log\left|E^*\right|_{p,i}$ is the logarithm of the calculated $\left|E^*(\omega)\right|$; $\log\varphi_{m,i}$ is the logarithm of the experimental value of $\varphi(\omega)$; $\log\varphi_{p,i}$ is the phase The logarithmic value of $\varphi(\omega)$.

3.3.2 Construction of the master curves of $E'(\omega)$ and $E''(\omega)$

Like the $\left|E^*(\omega)\right|$,the shape of the master curve of $E'(\omega)$ can also be described by the GSM. Therefore, the master curve of $E''(\omega)$ is constructed using Eq. (10)[22].

$$\log E'(f_r)=\delta'+\frac{\alpha'}{\left(1+\lambda' e^{\beta'+\gamma'(\log f_r)}\right)^{\frac{1}{\lambda'}}} \tag{10}$$

Through the approximate K-K relationship between $E'(\omega)$ and $E''(\omega)$ in equation (11), the master curve model of $E''(\omega)$ represented by equation (12) is derived.

$$E''\approx\frac{\pi}{2}\frac{\mathrm{dE}'(\omega)}{\mathrm{d}\ln(\omega)} \tag{11}$$

$$\begin{aligned}\log E''(f_r)=&\log\left(-\frac{\pi}{2}\alpha'\gamma'\right)+\left[\beta'+\gamma'\log f_r\right]\log e\\&-\left(1+\frac{1}{\lambda'}\right)\log\left[1+\lambda' e^{\beta'+\gamma'\log fr}\right]+\delta'+\frac{\alpha'}{\left(1+\lambda' e^{\beta'+\gamma'(\log f_r)}\right)^{\frac{1}{\lambda'}}}\end{aligned} \tag{12}$$

Selecting 20°C as the reference temperature, the model parameters of $E'(\omega)$ and $E''(\omega)$ are simultaneously solved by minimizing the parameter error. The error function is

$$error=\frac{1}{n}\sqrt{\sum_{i=1}^{n}\left(\frac{\log E'_{m,i}-\log E'_{p,i}}{\log E'_{m,i}}\right)^2}+\frac{1}{n}\sqrt{\sum_{i=1}^{n}\left(\frac{\log E''_{m,i}-\log E''_{p,i}}{\log E''_{m,i}}\right)^2} \tag{13}$$

where n is the total data points of the test, $n=18$; $\log E'_{m,i}$ is the logarithm of the experimental value of $E'(\omega)$; $\log E'_{p,i}$ is the logarithm of the calculated value of $E'(\omega)$; $\log E''_{m,i}$ is the logarithm of the experimental value of $E''(\omega)$; $\log E''_{p,i}$ is the logarithm of the calculated value of $E''(\omega)$.

3.4 Determining the discrete spectrum models

As the most commonly used discrete spectrum, the Prony series model is widely used to characterize the linear viscoelastic behavior of asphalt mixtures. The discrete spectrum composed of the coefficients of the Prony series contains all the viscoelastic information. The discrete spectrum can organically combine the viscoelastic parameters in different domains, and then realize the transformation of the master curve of the viscoelastic parameters.

3.4.1 Determining the discrete relaxation spectrum

According to the Boltzmann superposition principle, the integral constitutive relation of the $E(t)$ and $J(t)$ is[23]

$$\int_0^t E(t-\tau)\frac{\mathrm{d}J(\tau)}{\mathrm{d}\tau}\mathrm{d}\tau \tag{14}$$

From the GMM and the GKM, the Prony series expression of $E(t)$ and $J(t)$ can be deduced as follows[24]:

$$E(t)=E_e+\sum_{i=1}^{m}E_i e^{-(t/\rho_i)} \tag{15}$$

$$J(t) = J_g + \sum_{j=1}^{n} J_j \left(1 - e^{\left(-t/\tau_j\right)}\right) \tag{16}$$

where E_e is the equilibrium modulus; E_i is the relaxation strength; ρ_i is the relaxation time; J_g is the glassy compliance; J_j is the retardation strength; τ_j is the retardation time; and *t* is the test time.

Using the Carson transform, the following relationship can be obtained from equations (14)-(16)[25]:

$$\tilde{E}(s)\tilde{J}(s) = 1 \tag{17}$$

where $\tilde{E}(s)$ is the operator modulus, $\tilde{J}(s)$ is the operator compliance*;* *s* is the Lapace transform operator.

Since the complex function pair of viscoelastic material is excited by a sine wave. Therefore, the relationship between it and the operator function in the frequency domain is[8]

$$E^*(\omega) = \tilde{E}(s)\Big|_{s\to i\omega} \tag{18}$$

$$J^*(\omega) = \tilde{J}(s)\Big|_{s\to i\omega} \tag{19}$$

where $J^*(\omega)$ is the complex compliance .

According to (18) and (19), it can be obtained that $E^*(\omega)$ and $J^*(\omega)$ are reciprocals of each other in the frequency domain[26].

$$E^*(\omega)J^*(\omega) = 1 \tag{20}$$

According to Eq.(15)-(20), the expressions of $E'(\omega)$, $E''(\omega)$, $J'(\omega)$ and $J''(\omega)$ can be obtained by using Carson transform as[27]:

$$E'(\omega) = E_e + \sum_{i=1}^{m} E_i \frac{\omega^2\rho_i^2}{\omega^2\rho_i^2 + 1} \tag{21}$$

$$E''(\omega) = \sum_{i=1}^{m} E_i \frac{\omega\rho_i}{\omega^2\rho_i^2 + 1} \tag{22}$$

$$J'(\omega) = J_g + \sum_{j=1}^{n} J_j \frac{1}{\omega^2\tau_j^2 + 1} \tag{23}$$

$$J''(\omega) = \sum_{j=1}^{n} J_j \frac{\omega\tau_j}{\omega^2\tau_j^2 + 1} \tag{24}$$

From Eq. (21) and (22), it can be known that under the specified relaxation time, E_e and E_i can be obtained by fitting the $E'(\omega)$ and $E''(\omega)$ at different frequencies. The obtained parameters are put into equation (15) to get the master curve of $J(t)$.

3.4.2 Determining the discrete retardation spectrum models

$E(t)$ and $J(t)$ satisfy the integral relationship shown in Eq. (14), and the Prony series

expressions of $E(t)$ and $J(t)$ are brought into this equation, and the discrete retardation spectrum can be obtained. Substituting Eq. (15) and (16) into equation (14), we get

$$\int_0^t \left(E_e + \sum_{i=1}^{m} E_i e^{-\left[(t-\tau)/\rho_i\right]} \right) \left(J_g \delta(\tau) + \sum_{j=1}^{n} \frac{J_j}{\tau_j} e^{-(t/\tau_j)} \right) \mathrm{d}\tau = 1 \tag{25}$$

Where $\delta(\tau)$ denotes the Dirac delta function.

Further operation of Eq. (25) can be converted into

$$A_{kj} J_j = B_k \tag{26}$$

Where

$$A_{kj} = \begin{cases} E_e \left(1 - e^{-(t_k/\tau_j)}\right) + \sum_{i=1}^{m} \frac{\rho_i E_i}{\rho_i - \tau_j} \left(e^{-(t_k/\rho_i)} - e^{-(t_k/\tau_j)} \right) & \text{when } \rho_i \neq \tau_j \\ or & \\ E_e \left(1 - e^{-(t_k/\tau_j)}\right) + \sum_{i=1}^{m} \frac{\rho_i E_i}{\tau_j} e^{-(t_k/\rho_i)} & \text{when } \rho_i = \tau_j \end{cases} \tag{27}$$

$$B_k = 1 - \left(E_e + \sum_{i=1}^{m} E_i e^{-(t_k/\rho_i)} \right) \Bigg/ \left(E_e + \sum_{i=1}^{m} E_i \right) \tag{28}$$

Where $k = 1, 2, \cdots, p$; $j = 1, 2 \cdots, n$; t_k is the upper limit of the integral of formula (14).

From Eq. (26)-(28), it can be seen that when the discrete relaxation spectrum and E_e are known and τ_j and t_k are set, the discrete retardation spectrum models can be obtained by solving the inhomogeneous linear equation system, and then $E(t)$ can be obtained.

3.5 Determining the continuous spectrum

3.5.1 Determining the continuous relaxation spectrum models

When the time interval of discrete spectrum is close to infinitely small, it will evolve into continuous spectrum. The conversion between the models of the viscoelastic parameters and the continuous spectrum can be realized by using the integral transformation theory. According to the internal relationship between the storage modulus model (ie the GSM) and the continuous relaxation spectrum models $H(\rho)$, the functional relationship of $H(\rho)$ on the model of $E'(\omega)$ can be deduced.

The integral relationship between $E(t)$ and the $H(\rho)$ is[28]

$$E(t) = E_e + \int_{-\infty}^{+\infty} H(\rho) e^{-(t/\rho)} \mathrm{d}\ln\rho \tag{29}$$

According to the Eq. (18), the integral expression of the $E^*(\omega)$ with respect to $H(\rho)$ is obtained by calculating[28]:

$$E^*(\omega) = E_e + \int_{-\infty}^{+\infty} H(\rho) \frac{\omega^2 \rho^2 + i\omega\rho}{\omega^2 \rho^2 + 1} \mathrm{dln}\rho \tag{30}$$

Separating the real and imaginary parts of Eq.(30), the integral relation of the $E'(\omega)$ and $E''(\omega)$ with respect to the time spectrum is obtained as

$$E'(\omega) = E_e + \int_{-\infty}^{+\infty} H(\rho) \frac{\omega^2 \rho^2}{\omega^2 \rho^2 + 1} \mathrm{dln}\rho \tag{31}$$

$$E''(\omega) = \int_{-\infty}^{+\infty} H(\rho) \frac{\omega \rho}{\omega^2 \rho^2 + 1} \mathrm{dln}\rho \tag{32}$$

Applying Stieltjes and its inverse transform to Eq. (31), $H(\rho)$ is obtained with respect to $E'(\omega)$ as

$$H(\rho) = \pm \frac{2}{\pi} \mathrm{Im} \left\{ E' \left[\rho^{-1} \exp\left(\pm \frac{\pi}{2} i \right) \right] \right\} \tag{33}$$

The model of $E'(\omega)$ is written in non-logarithmic form and replacing ω with $\rho^{-1} \exp\left(\pm \frac{\pi}{2} i \right)$ substituted into Eq. (33) to obtain the expression of $H(\rho)$ after integral transformation and simplification

$$H(\rho) = \pm \frac{\pi}{2} \mathrm{Im} \left\langle \exp(A) \exp\left\{ \frac{B}{\left[1 + \lambda \bullet \exp\left(C + D \ln \frac{\alpha_T}{\rho} \right) \bullet \exp\left(\pm \frac{\pi D}{2} i \right) \right]^{\frac{1}{\lambda}}} \right\} \right\rangle \tag{34}$$

Where $A = \delta \ln(10)$; $B = \alpha \ln(10)$; $C = \beta - \gamma \log(2\pi)$; $D = \frac{\gamma}{\ln(10)}$ 。

Using Euler's equation, the complex exponential function of Eq. (34) is converted into a trigonometric function, and the expression of $H(\rho)$ is obtained by further solving

$$H(\rho) = \pm \frac{\pi}{2} \mathrm{Im} \left\{ \exp\left[P(\rho) \right]^{\frac{1}{\lambda}} \right\} \tag{35}$$

Separating $P(\rho)$ into $X(\rho)$ and $Y(\rho)$, the expressions of the three are

$$P(\rho) = X(\rho) \mp Y(\rho) \tag{36}$$

$$X(\rho) = \frac{B^{\lambda} \left[1 + \lambda \exp(E) \cos \frac{\pi D}{2} \right]}{1 + \lambda^2 \exp(2E) + 2\lambda \exp(E) \cos \frac{\pi D}{2}} \tag{37}$$

$$Y(\rho)=\frac{B^{\lambda}\left[1+\lambda\exp(E)\sin\frac{\pi D}{2}\right]}{1+\lambda^{2}\exp(2E)+2\lambda\exp(E)\cos\frac{\pi D}{2}} \tag{38}$$

Where $E=C+D\ln\left(\frac{\alpha_T}{\rho}\right)$ 。

Let $F(\rho)=X^2(\rho)+Y^2(\rho)$ and $G=\arctan\frac{Y(\rho)}{X(\rho)}$, use Euler's formula again to further simplify the formula, and finally obtain the relationship between $H(\rho)$ and the parameters of the model of $E'(\omega)$ as

$$H(\rho)=-\frac{2}{\pi}\exp\left(A+F^{\frac{1}{2\lambda}}\cos\frac{G}{\lambda}\right)\sin\left(F^{\frac{1}{2\lambda}}\sin\frac{G}{\lambda}\right) \tag{39}$$

3.5.2 Determining the Continuous retardation Spectrum

As shown in Eq. (40), the retardation spectrum function can be derived from the known relaxation spectrum function[28]. In order to ensure a one-to-one correspondence between the retardation time and the relaxation time during calculation, the retardation time is also represented by τ .

$$L(\tau)=\frac{H(\tau)}{Z^2(\tau)+\pi^2H^2(\tau)} \tag{40}$$

$$Z(\tau)=E_e+\int_0^{+\infty}\frac{H(u)}{u-\tau}du \tag{41}$$

4. Results and Discussion

4.1 Linear viscoelasticity characterization of basalt fiber asphalt mixture

4.1.1 Master curves of $|E^*(\omega)|$ and $\varphi(\omega)$

Use Excel's Solver function to calculate equation (9) to obtain the model parameters of $|E^*(\omega)|$ and $\varphi(\omega)$. The fitting results are shown in Table 1, and the master curves of $|E^*(\omega)|$ and $\varphi(\omega)$ are shown in Figures 2 and 3.

Table 1 Models parameters of $|E^*(\omega)|$ and $\varphi(\omega)$

Types of asphalt mixtures	Parameters							Error (%)
	δ	α	λ	β	γ	C_1	C_2	
BF-0	1.95	2.77	0.76	0.02	-0.48	15.85	200.43	5.77
BF-1	2.21	2.41	0.84	-0.03	-0.64	23.93	325.36	5.46
BF-2	2.36	2.13	0.83	0.04	-0.70	24.27	351.58	5.20
BF-3	2.08	2.57	0.88	-0.11	-0.58	28.48	350.00	4.25

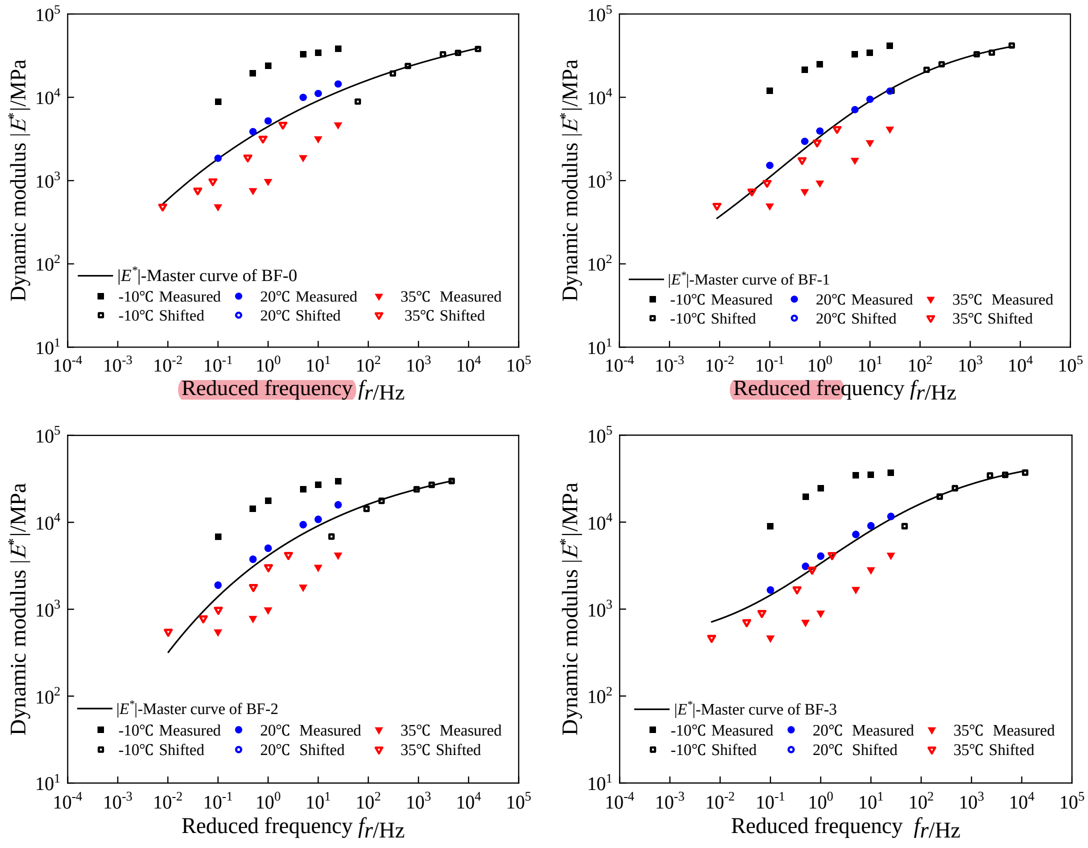

Fig. 2 Master curves of $|E^*(\omega)|$

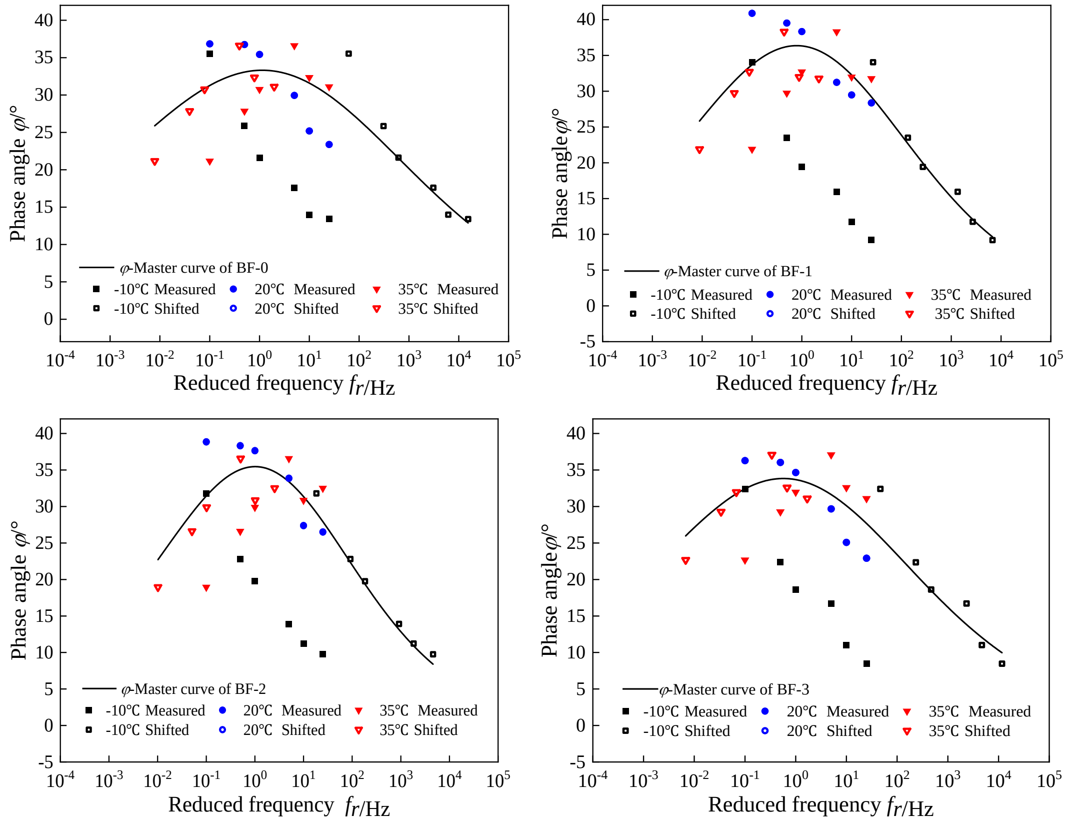

Fig. 3 Master curves of $\varphi(\omega)$

To compare the effects of basalt fiber content on the master curve of $|E^*(\omega)|$ and $\varphi(\omega)$, the

two master curves of viscoelastic parameters are constructed in Fig. 4(a) and Fig. 4(b), respectively. At high frequency, the dynamic modulus of BF-2 is the smallest, the dynamic modulus of BF-0 is the largest, and the dynamic modulus of BF-0 and BF-3 are both smaller than BF-0. At low frequency, the dynamic modulusof BF-1, BF-2 and BF-3 are all larger than BF-0, and the dynamic modulus of the three groups of basalt fiber asphalt mixtures have little difference. According to TTPS, it shows that the basalt fiber asphalt has good mixed deformation ability and strong toughness at low-temperature, and can better resist the action of external loads and disperse the stress.

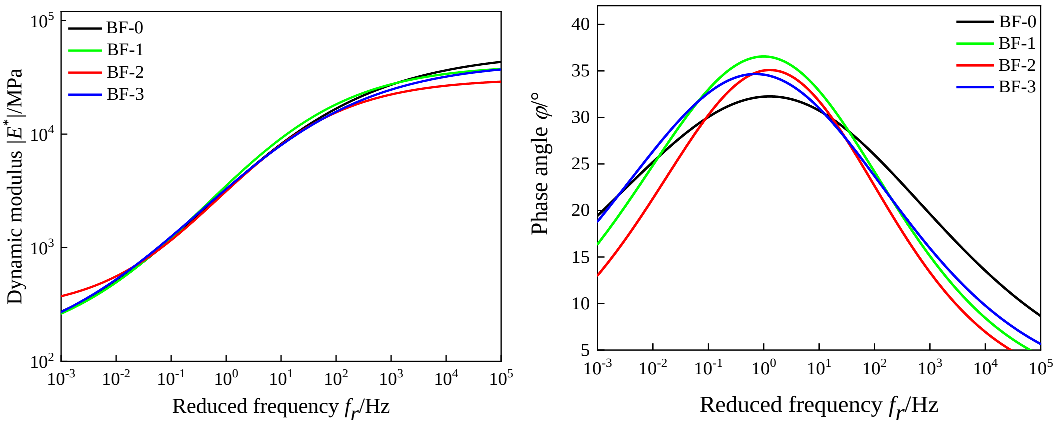

(a) Comparison the master of $\left|E^*(\omega)\right|$ (b) Comparison the master curves of $\varphi(\omega)$

Fig. 4 Comparison the master curves of $\left|E^*(\omega)\right|$ and $\varphi(\omega)$

Under high frequency and low frequency conditions ,the phase angles of BF-1, BF-2 and BF-3 are lower than BF-0; among them, BF-2 has the smallest phase angle. This is because the basalt fibers adsorb the asphalt, which reduces the viscoelasticity ratio of the asphalt mixture, and the fibers form a grid structure in the asphalt mixture, which constrains the deformation of the asphalt mixture and reduces the phase angle. This is because basalt fibers improves the low-temperature deformation capacity of asphalt mixtures by changing their the internal structure.

4.1.2 Master curves of $E'(\omega)$ and $E''(\omega)$

The model parameters of $E'(\omega)$ and $E''(\omega)$ are simultaneously obtained by calculating Eq.(13). The fitting results are shown in Table 3, and the master curves of $E'(\omega)$ and $E''(\omega)$ are shown in Figures (5) and (6).

Table 2 Models Parameters of $E'(\omega)$ and $E''(\omega)$.

Types of asphalt mixtures	Parameters							Error (%)
	δ'	α'	λ'	β'	γ'	C_1'	C_2'	
BF-0	2.17	2.70	0.88	0.07	-0.60	20.72	274.93	1.03
BF-1	2.37	2.39	0.33	-0.11	-0.62	28.30	381.81	1.39
BF-2	2.50	2.13	0.33	-0.03	-0.65	26.59	382.06	1.70
BF-3	2.29	2.48	0.24	-0.14	-0.53	19.89	250.94	1.14

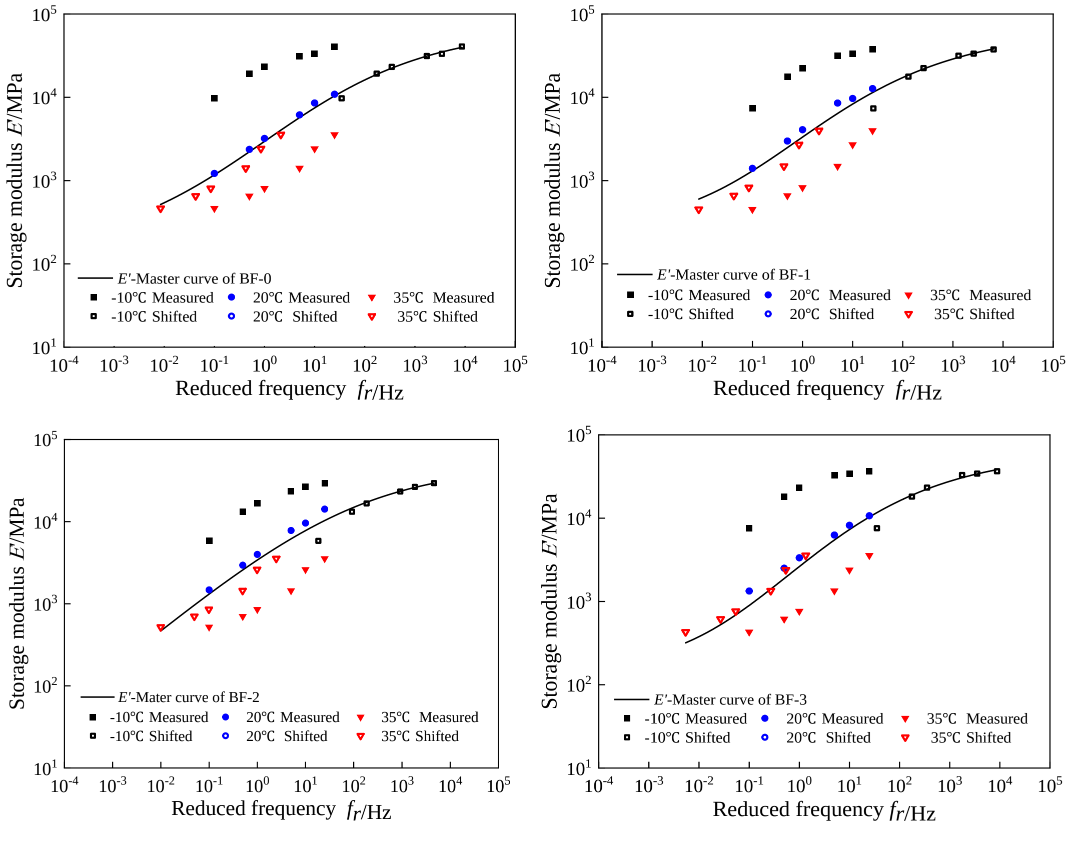

Fig.5 Master curves of $E'(\omega)$

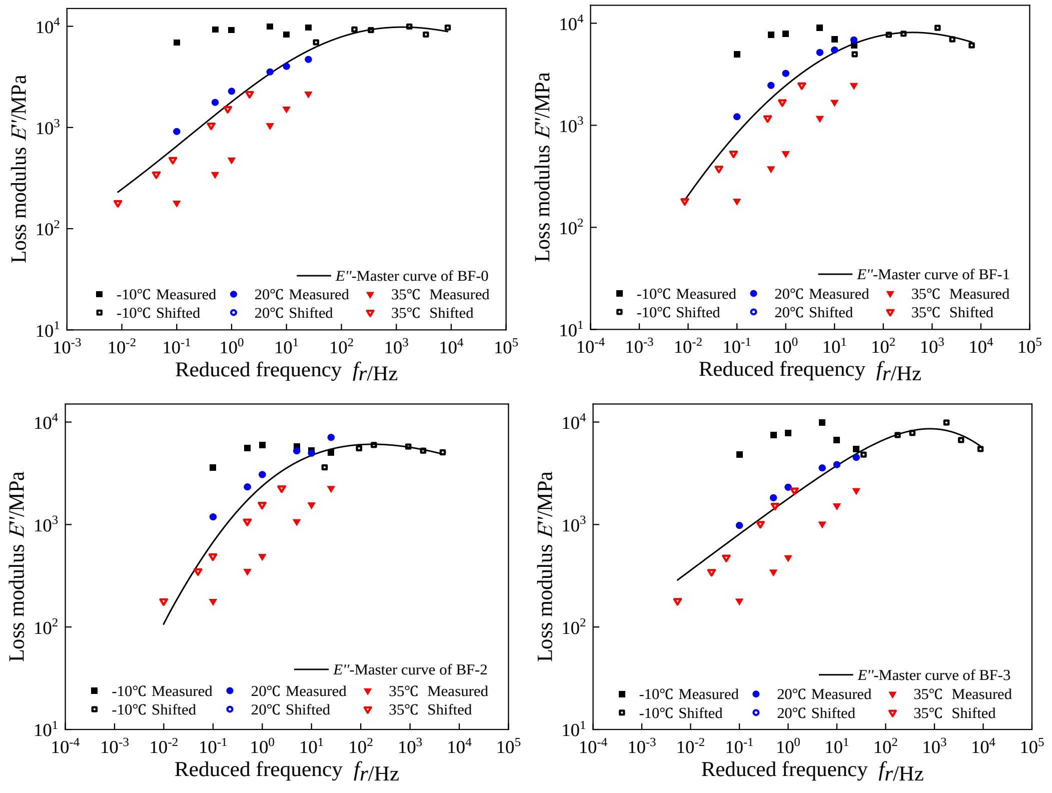

Fig.6 Master curves of $E''(\omega)$

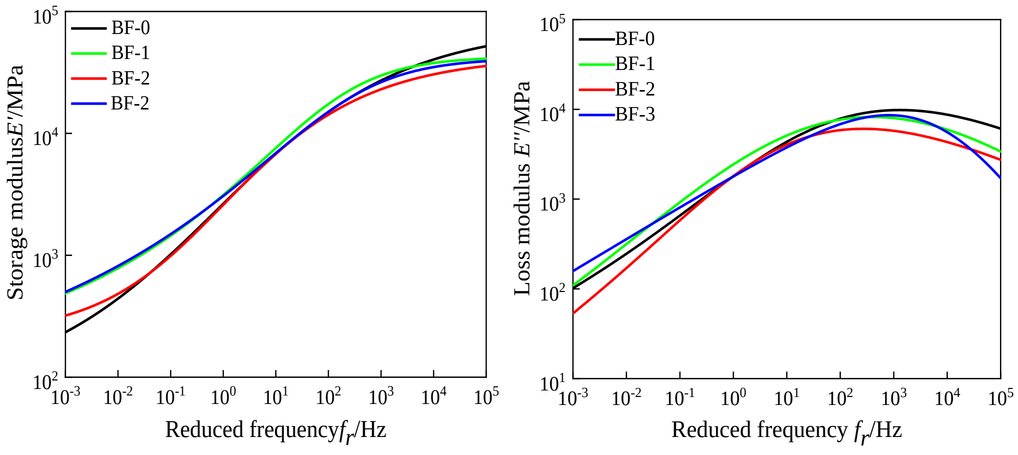

(a) Comparison the master of $E'(\omega)$ (b) Comparison the master curves of $E''(\omega)$

Fig. 7 Comparison the master curves of $E'(\omega)$ and $E''(\omega)$

At high frequency, the storage modulus first decreases and then increases with the increase of basalt fiber content, and the loss modulus decreases with the increase of basalt fiber content. At low frequency, the storage modulus also increases with the increase of basalt fiber content, and the loss modulus first decreases and then increases with the increase of basalt fiber content. It shows that at low temperature, adding basalt fiber can reduce the energy stored and lost in the asphalt mixture, and correspondingly reduce its low-temperature elastic capacity and low-temperature viscous deformation.

4.2 Construction of master curves of $E(t)$ and $J(t)$ from discrete spectrum

4.2.1 Determining the discrete relaxation spectrum

A discrete relaxation spectrum containing all viscoelastic information was obtained using or the collocation method. To reduce the fluctuation of the curve, the relaxation time are taken at intervals of 1 on the logarithmic time axis. Using Equation (42) to minimize the target error, the calculated Prony series results of the relaxation modulus are shown in Table 4.

$$error = \frac{1}{n}\sqrt{\sum_{i=1}^{n}\left(\frac{E'_{gs,i} - E'_{ps,i}}{E'_{gs,i}}\right)^2} + \frac{1}{n}\sqrt{\sum_{i=1}^{n}\left(\frac{E''_{gs,i} - E''_{ps,i}}{E''_{gs,i}}\right)^2} \tag{42}$$

Where n is the number of data points, $n=18$; $E'_{gs,i}$ is the estimated value of the storage modulus master curve; $E'_{ps,i}$ is the storage modulus Prony series calculation value; $E''_{gs,i}$ is the loss modulus master curve estimate value; $E''_{ps,i}$ is the calculated value of the loss modulus Prony series.

Table 3 Prony series coefficients of $E(t)$

i	ρ_i	E_i			
		BF-0	BF-1	BF-2	BF-3
1	2.00E-07	2003.47	2007.60	1990.01	1752.87
2	2.00E-06	3288.98	3270.51	3094.23	2869.47
3	2.00E-05	10362.20	8848.07	7263.62	10359.91
4	2.00E-04	14549.99	12328.44	8601.48	11342.64
5	2.00E-03	10263.25	11284.45	9341.45	9963.79
6	2.00E-02	5496.59	6596.66	5678.70	5287.52
7	2.00E-01	2170.16	2265.82	2130.64	1958.89
8	2.00E+00	679.75	616.08	512.75	645.95

9	2.00E+01	214.88	144.69	138.20	161.07
10	2.00E+02	90.52	88.04	36.66	81.63
11	2.00E+03	50.19	40.32	34.56	40.94
12	2.00E+04	54.32	42.84	42.33	25.84
13	2.00E+05	34.86	12.57	18.61	21.62
14	2.00E+06	13.34	2.95	9.05	11.62
15	2.00E+07	13.34	2.95	9.05	11.62
E_e		161.00	218.33	312.02	176.15

4.2.2 Derivation of discrete retardation spectrum from discrete relaxation spectrum

The discrete retardation spectrum models can be derived using equations (26)-(28). This process generally requires three steps, namely determining the retardation time and t_k ,calculating the retardation intensity and determining the glassy compliance.

First, the discrete delay time can be calculated by Equation (43). The roots of Equation (43) can be quickly obtained by using the graphical root-finding method. Taking the absolute value of $\tilde{E}(s)$ as the ordinate and -1/s as the abscissa, calculate 1500 data points and draw a graph. The distribution curve of $\tilde{E}(s)$ and -1/s is shown in Figure 8. The abscissa of the minimum value in the figure is the discrete delay time.

$$\lim_{s \to -(1/\tau_j)} \tilde{E}(s) = 0 \tag{43}$$

After determining the retardation time τ_j $(j = 1, 2, \cdots, n)$, the next step is to determine t_k $(k = 1, 2, \cdots, p)$ .Solving the inhomogeneous system of linear equations requires $p > n$ because of $\rho_i \neq \tau_j$, and setting $t_k = 10^{[(k-1)/2]-7}$ $(k = 1, 2 \cdots, 30)$.

Afterwards, the retardation strength is determined by solving the error function equation (26).

$$error = \frac{1}{N} \sqrt{\sum_{i=1}^{N} \left(\frac{B_i - \left(A_{kj} J_j \right)_i}{B_i} \right)^2} \tag{44}$$

Where N is the total number of data points, N=30.

Finally, according to the initial-value and final-value theorems of Laplace transform, the glassy compliance is determined using Eq. (45)

$$J_g = \lim_{t \to 0} J(t) = \lim_{s \to \infty} \tilde{J}(t) = \frac{1}{\lim_{s \to \infty} \tilde{E}(s)} = \frac{1}{\lim_{t \to 0} E(t)} = \frac{1}{E_e + \sum_{i=1}^{m} E_i} \tag{45}$$

Table 5 shows the coefficients of the creep compliance Prony series obtained by the transformation of the relaxation spectrum of the four asphalt mixtures.

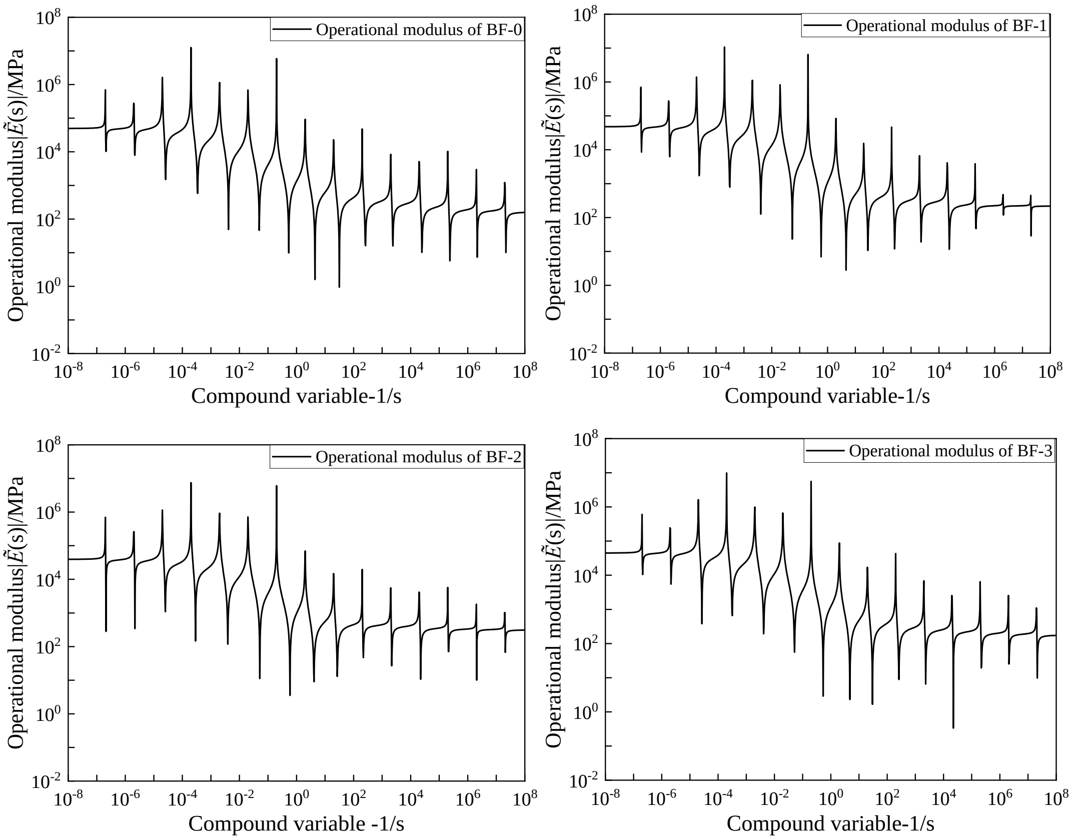

Fig.8 Distribution curve of $\tilde{E}(s)$ with -1/s

Table 4 Prony series coefficients of $J(t)$

j	BF-0		BF-1		BF-2		BF-3	
	τ_j	J_j	τ_j	J_j	τ_j	J_j	τ_j	J_j
1	2.11E-07	7.09E-07	2.11E-07	6.59E-07	2.11E-07	2.36E-07	2.11E-07	1.03E-06
2	2.18E-06	2.05E-06	2.18E-06	2.26E-06	2.18E-06	4.44E-06	2.18E-06	8.82E-07
3	2.60E-05	5.40E-06	2.48E-05	4.35E-06	2.54E-05	6.17E-06	2.60E-05	1.04E-05
4	3.44E-04	1.74E-05	3.12E-04	1.71E-05	2.89E-04	1.60E-05	3.04E-04	1.44E-05
5	4.12E-03	5.48E-05	4.02E-03	3.76E-05	3.92E-03	4.46E-05	3.82E-03	4.12E-05
6	4.93E-02	1.14E-04	5.44E-02	1.29E-04	5.17E-02	1.38E-04	4.93E-02	1.16E-04
7	5.34E-01	3.75E-04	5.90E-01	4.21E-04	5.90E-01	4.58E-04	5.21E-01	3.25E-04
8	4.42E+00	8.80E-04	4.65E+00	1.09E-03	4.11E+00	8.67E-04	5.00E+00	7.89E-04
9	3.16E+01	1.03E-03	2.79E+01	9.55E-04	2.66E+01	5.99E-04	4.24E+01	1.92E-03
10	2.62E+02	8.53E-04	2.55E+02	7.40E-04	2.20E+02	3.17E-04	3.78E+02	2.67E-03
11	2.39E+03	6.70E-04	2.27E+03	4.59E-04	2.17E+03	2.06E-04	2.77E+03	2.69E-03
12	2.47E+04	5.48E-04	2.35E+04	2.05E-04	2.24E+04	1.55E-04	3.23E+04	4.18E-03
13	2.37E+05	3.01E-04	2.15E+05	1.56E-04	2.15E+05	1.06E-04	2.43E+05	2.88E-03
14	2.16E+06	1.86E-04	2.06E+06	1.02E-04	2.06E+06	8.60E-05	2.06E+06	2.33E-03
15	2.18E+07	2.70E-05	2.02E+07	2.61E-05	2.07E+07	9.22E-05	2.02E+07	3.04E-04
J_g	2.02E-05		2.09E-05		2.55E-05		2.24E-05	

The discrete relaxation and retardation spectrum are drawn according to Table 4 and Table 5, as shown in Figure 9 and 10. Both discrete spectrum have obvious asymmetry. And with the increase of basalt fiber content, the peak relaxation strength showed a trend of decreasing first, while the discrete retardation spectrum did not have a specific change rule.

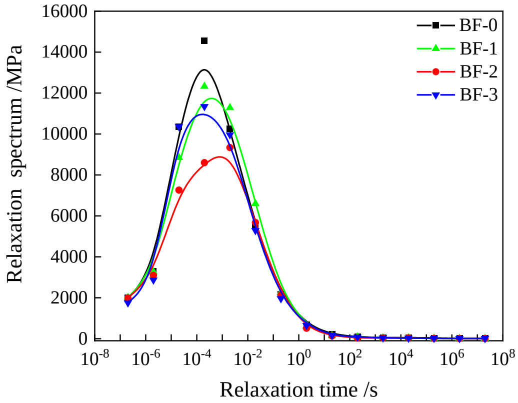

Fig.9 Discrete relaxation spectrum

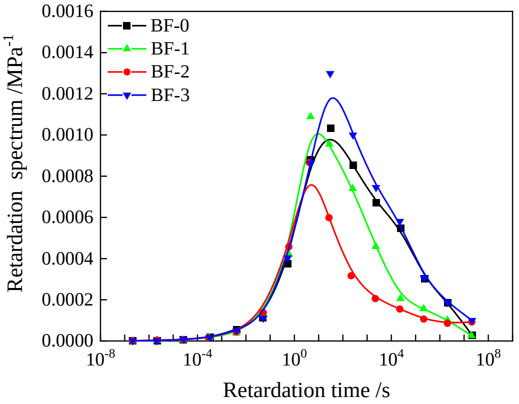

Fig.10 Discrete retardation spectrum

4.2.3 Construction the master curves of $E(t)$ and $J(t)$ from discrete spectrum

The Prony series parameter of relaxation modulus in Table 4 is put into Eq.(15), and the mater curve of relaxation modulus can be obtained by conversion, as shown in Fig.11. And by putting the creep compliance Prony series parameters in Table 5 into Eq.16, and the relaxation modulus master curve of creep compliance can be constructed, as shown in Fig.12.

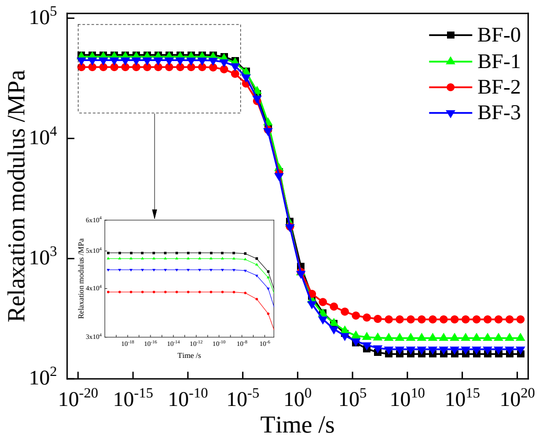

Fig.11 Master curve of $E(t)$ from discrete relaxation spectrum

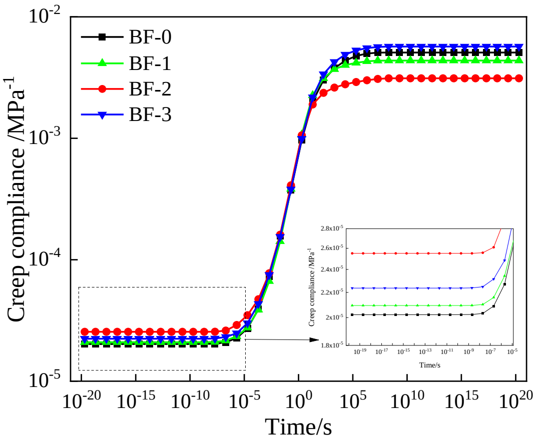

Fig.12 Master curve of $J(t)$ from discrete retardation spectrum

The relaxation modulus reaches its maximum value when the loading time is short. When the loading time is very long, the relaxation modulus reaches the minimum value. And the relaxation modulus was found to vary greatly from 10^{-5} to 10^{5}. This is because, the internal stress of the asphalt mixture is accumulated for a short loading time. With the extension of the loading time, the internal stress dissipates rapidly, and a small amount of stress remains after a long time of relaxation. At the same time, in a short loading time, the relaxation modulus decreased with the increase of basalt fiber content and then increased, and the relaxation modulus was the smallest when the basalt fiber content was 0.2%. It shows that basalt fiber can improve the low-temperature relaxation ability and the thermal shrinkage cracking resistance of asphalt mixtures, and reduce the accumulation of temperature stress of asphalt mixture. This is because basalt fiber improves the stress diffusion ability of the asphalt mixture, thereby improving the low-temperature crack resistance. In a long loading time, the relaxation modulus first increased and then decreased with the increase of basalt

fiber content, and the relaxation modulus reached the maximum when the basalt fiber content was 0.2%.

When the loading time is short, the creep compliance tends to the minimum value; when the loading time is very long, the creep compliance tends to the maximum value, and the creep compliance changes the largest from 10^{-5} to 10^{5} s . This phenomenon can be attributed to the softening of asphalt at high-temperature. As a whole, the asphalt mixture is composed of additives such as coarse and fine aggregates, asphalt, and mineral powder. With the increase of temperature or loading time, the viscosity of asphalt increases. The effect of the knot is weakened, which leads to a reduction in the overall resistance to deformation of the asphalt. At the same time, in the short loading time, the creep compliance first increases and then decreases with the basalt fiber content, and the creep compliance of BF-2 is the largest. For a longer loading time, the creep compliance decreases and then increases with the increase of the basalt fiber content, and the creep compliance of BF-2 is the smallest. This shows that basalt fiber can improve the deformation ability and resist creep deformation at high temperature of the asphalt mixture. And correspondingly improve its anti-rutting ability. Moreover, when the basalt fiber content is 0.2%, the effect of improving the deformation resistance of the asphalt mixture is the best.

4.3 Construction master curves of $E(t)$ and $J(t)$ from Continuous spectrum

4.3.1 Determining the continuous relaxation Spectrum

The model parameters of $E'(\omega)$ and $E''(\omega)$ in Table 3 are put into Eq.(39), and the continuous relaxation spectrum model of four kinds of asphalt mixture can be obtained. Taking the relaxation time as the abscissa and $H(\rho)$ as the ordinate, the continuous relaxation spectrum when the reference temperature is 20°C is obtained, as shown in Figure 13. The relaxation spectrum is asymmetric. And the peak of the continuous spectrum is the same as that obtained by the discrete relaxation time spectrum. The parametric instruments used were derived from master curves of $E'(\omega)$ and $E''(\omega)$. The constructed continuous relaxation spectrum therefore contains all the linear viscoelastic information.

4.3.2 Constructed the continuous retardation spectrum from the continuous relaxation spectrum

When deriving the continuous retardation spectrum from Eq.(40), the integral term $Z(\tau)$ and the equilibrium modulus E_e need to be calculated. Use the chained trapezoidal rule to solve the integral term $Z(\tau)$ .The integration interval of τ is selected as $\left[10^{-40}, 10^{30}\right]$, the integration interval of u is selected as $\left[10^{-40.0025}, 10^{29.9975}\right]$, the number of sub-intervals is 14000, and the length of the sub-interval is $\log\Delta\tau = \log\Delta u = 0.005$. The equilibrium modulus E_e can be calculated from equation (46).

$$E_e = E'(\omega = 0) = E'(f_r = 0) = 10^{\delta} \tag{46}$$

The numerical expression of $Z(\tau)$ is

$$Z(\tau)=10^{\delta}+(\ln 10)\cdot\frac{0.005}{2}\cdot\left\{\begin{array}{l}\frac{10^{-40.0025}}{10^{-40.0025}-\tau}H\left(10^{-40.0025}\right)\\+2\left[\frac{10^{-40.0025}}{10^{-40.0025}-\tau}H\left(10^{-40.0025}\right)+\cdots+\frac{10^{29.9925}}{10^{29.9925}-\tau}H\left(10^{29.9925}\right)\right]\\+\frac{10^{29.9975}}{10^{29.9975}-\tau}H\left(10^{29.9975}\right)\end{array}\right\} \tag{47}$$

Substituting the discretized values of $Z(\tau)$ and $H(\tau)$ into formula (40) can obtain the continuous retardation spectrum values of the four asphalt mixtures, and generate the continuous retardation spectrum shown in Fig. 14 . As shown in Fig. 14, the variation law of the peak retardation intensity of the continuous retardation spectrum with the fiber content is the same as that of the discrete retardation spectrum. However, the continuous retardation spectrum has lower peak retardation intensity than the discrete retardation spectrum.

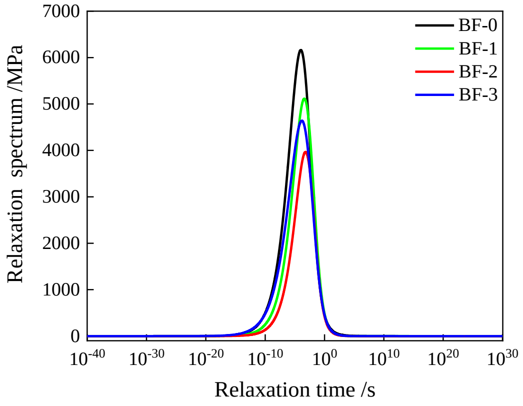

Fig. 13 Continuous relaxation spectrum of asphalt mixtures

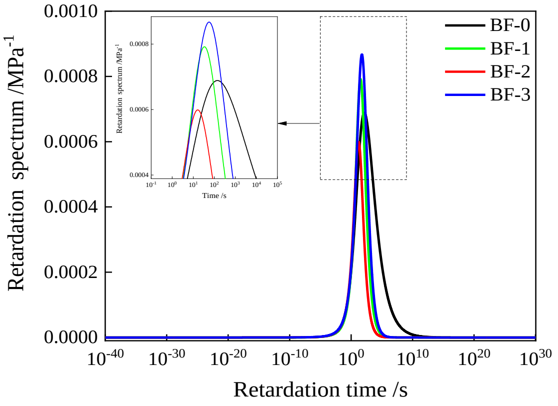

Fig. 14 Continuous retardation spectrum of asphalt mixtures

4.3.3 Constructed master curves of $E(t)$ and $J(t)$ based on continuous spectrum

Use the chained trapezoidal rule to solve Eq. (29), the integration interval is selected as $\left[10^{-40},10^{30}\right]$, and the subinterval length is selected as $\log\Delta\rho=0.005$. The numerical expression of $E(t)$ is shown in Eq. (48). The models of $E(t)$ of the four asphalt mixtures were calculated according to Eq.(48), and construct the master curves， as shown in Fig. 15. The master curves of $E(t)$ obtained from continuous and discrete relaxation spectrum have the same trend of variation with loading time. Moreover, at short and long loading times, the master curves of $E(t)$ with the basalt fiber content has the same trend, also.

$$E(t) \approx E_e + (\ln 10) \cdot \frac{0.005}{2} \cdot \left\{ \begin{array}{l} H\left(10^{-40}\right) e^{-\left(t/10^{-40}\right)} \\ +2\left[H\left(10^{-39.995}\right) e^{-\left(t/10^{-39.995}\right)} + \cdots + H\left(10^{29.995}\right) e^{-\left(t/10^{29.995}\right)} \right] \\ +H\left(10^{30}\right) e^{-\left(t/10^{30}\right)} \end{array} \right\} \tag{48}$$

The model of $J(t)$ can be calculated from equation (49). Calculate Eq.(49) using the same method and settings as for relaxation modulus. The numerical expression of $J(t)$ with respect to $L(\tau)$ is shown in Eq. (50).

$$J(t) = J_g + \int_{-\infty}^{+\infty} L(\tau)\left(1 - e^{-(t/\tau)}\right) \mathrm{d}\ln\tau \tag{49}$$

$$J(t) \approx J_g + (\ln 10) \cdot \frac{0.005}{2} \cdot \left\{ \begin{array}{l} L\left(10^{-40}\right)\left(1 - e^{-\left(t/10^{-40}\right)}\right) \\ +2\left[L\left(10^{-39.995}\right)\left(1 - e^{-\left(t/10^{-39.995}\right)}\right) + \cdots + L\left(10^{29.995}\right)\left(1 - e^{-\left(t/10^{29.995}\right)}\right) \right] \\ +L\left(10^{30}\right)\left(1 - e^{-\left(t/10^{30}\right)}\right) \end{array} \right\} \tag{50}$$

In Eq.(50), there is still one unknown J_g, and according to the initial-value and final-value theorem of Laplace transform J_g and $E'(\omega)$ have the relationship shown in Eq. (51).

$$J_g = J(0) = \frac{1}{E(0)} = \frac{1}{E'(\omega \to \infty)} = \frac{1}{10^{\delta+\alpha}} \tag{51}$$

According to equation (50) and (51), the master curve of $J(t)$ can be construct, as shown in Fig.16. The master curves of $J(t)$ obtained from the continuous and discrete retardation spectrum has the same trend with loading time. Moreover, the creep compliance changes with the basalt fiber content in the same trend in short loading time, also. However, the creep compliance of BF-3 is larger than that of Bf-2 in a very short loading time. And it is larger than that of BF-0 in a very long loading time.

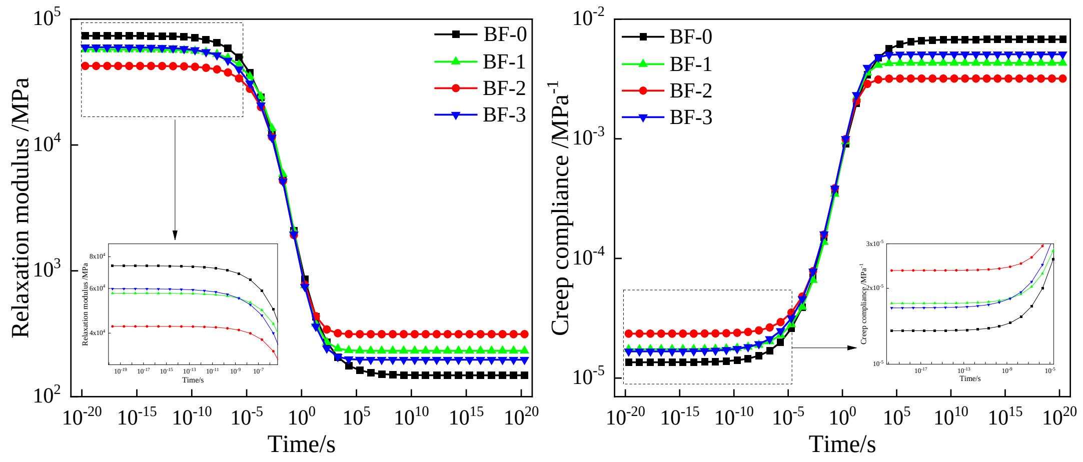

Fig. 15 Master curve of $E(t)$ from continuous relaxation spectrum

Fig. 16 Master curve of $J(t)$ from continuous retardation spectrum

4.4 Comparison of master curves differences of $E(t)$ and $J(t)$

To evaluate the difference between the master curves of $E(t)$ and $J(t)$ constructed from two types of spectrum models, the relative errors of $E(t)$ and $J(t)$ are calculated by equations (52) and (53). And the relative errors are established as a function of Load time change graph, as shown in Figures (17) and (18). In the range 10^{-5} to 10^{0} s, the errors calculated from two types of spectrum models are the smallest. But significant differences are shown in loading times less than 10^{-5} s. These reasons can be seen from the continuous and the discrete spectrum.

$$error_E = \frac{\left|E_C(t) - E_D(t)\right|}{E_C(t)} \times 100\% \tag{52}$$

$$error_J = \frac{\left|J_C(t) - J_D(t)\right|}{J_C(t)} \times 100\% \tag{53}$$

Where $E_C(t)$ and $J_C(t)$ are calculated the continuous relaxation and retardation spectrum, respectively; $E_D(t)$ and $J_D(t)$ are from the discrete relaxation and retardation spectrum, respectively.

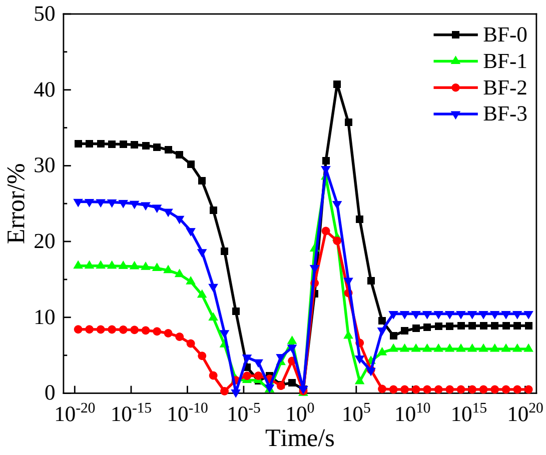

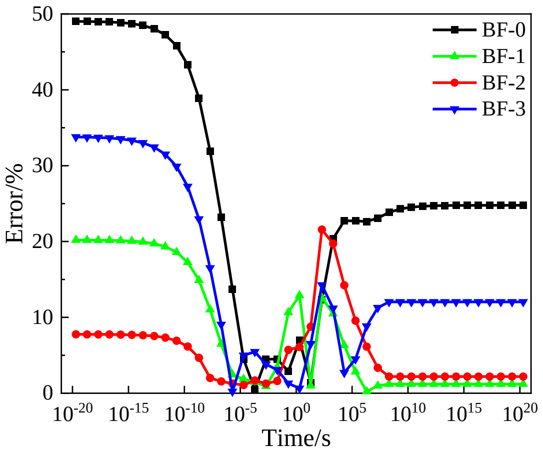

Fig.17 Relative errors of $E(t)$

Fig.18 Relative errors of $J(t)$

4.5 Accuracy check of the master curves of $E(t)$ and $J(t)$

The errors of the master curves of $E(t)$ and $J(t)$ mainly come from two aspects: on the one hand, the errors generated by using Eq. (10) and Eq. (12) to construct the master curves of $E'(\omega)$ and $E''(\omega)$; on the other hand, the error resulting from the transformation of the master curve of the viscoelastic parameters. Therefore, the accuracy of the master curves of $E(t)$ and $J(t)$ needs to be evaluated. Convert the master curves of

4.5.1 Accuracy verification of the master curves of $E(t)$

According to Eq. (21) and (22), E' and E'' of each of the four asphalt mixtures at the reduced frequency can be directly calculated.

When calculating E' and E'' from $H(\rho)$, Eq.(31) and (32) are calculated using the chained trapezoidal rule, the integral interval is selected as $\left[10^{-40},10^{30}\right]$, and the sub-interval length is selected as $\log\Delta\rho=0.005$. The numerical model expressions of s E' and E'' with respect to $H(\rho)$ are respectively:

$$E'(\omega)\approx 10^{\delta}+(\ln 10)\cdot\frac{0.005}{2}\cdot\left\{\begin{aligned}&H\left(10^{-40}\right)\frac{\omega^2\left(10^{-40}\right)^2}{1+\omega^2\left(10^{-40}\right)^2}\\&+2\left[H\left(10^{-39.995}\right)\frac{\omega^2\left(10^{-39.995}\right)^2}{1+\omega^2\left(10^{-39.995}\right)^2}+\cdots H\left(10^{29.995}\right)\frac{\omega^2\left(10^{29.995}\right)^2}{1+\omega^2\left(10^{29.995}\right)^2}\right]\\&+H\left(10^{30}\right)\frac{\omega^2\left(10^{30}\right)^2}{1+\omega^2\left(10^{30}\right)^2}\end{aligned}\right\} \tag{54}$$

$$E''(\omega)\approx(\ln 10)\cdot\frac{0.005}{2}\cdot\left\{\begin{aligned}&H\left(10^{-40}\right)\frac{\omega 10^{-40}}{1+\omega^2\left(10^{-40}\right)^2}+H\left(10^{-39.995}\right)\\&+2\left[H\left(10^{-39.995}\right)\frac{\omega 10^{-39.995}}{1+\omega^2\left(10^{-39.995}\right)^2}+\cdots+H\left(10^{29.995}\right)\frac{\omega 10^{29.995}}{1+\omega^2\left(10^{29.995}\right)^2}\right]\\&H\left(10^{30}\right)\frac{\omega 10^{30}}{1+\omega^2\left(10^{30}\right)^2}\end{aligned}\right\} \tag{55}$$

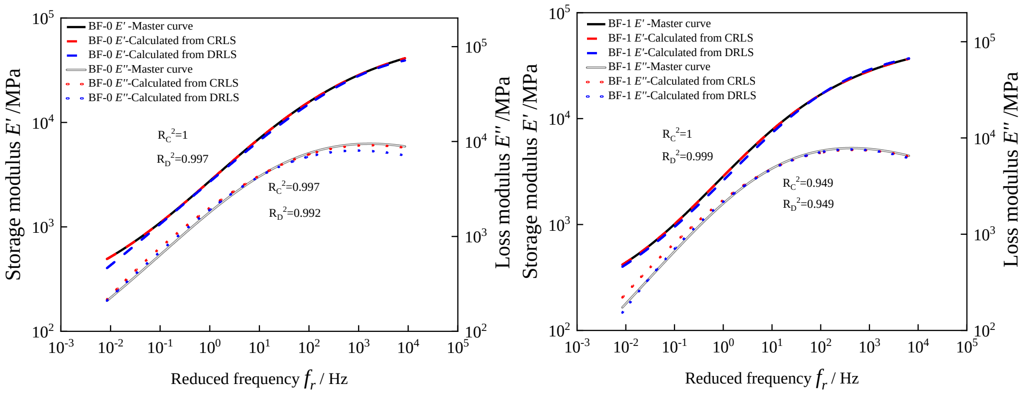

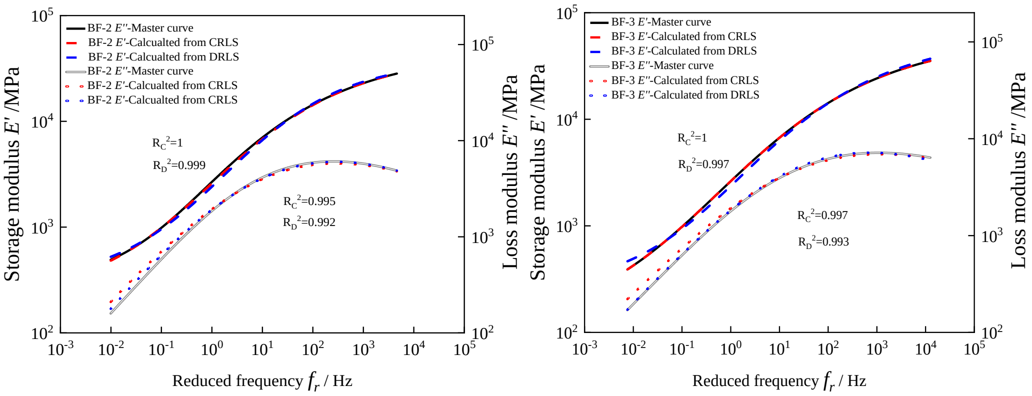

Fig. 19. Comparison of calculated E' and E'' with the master curves of $E'(\omega)$ and $E''(\omega)$

The storage modulus and loss modulus at each reduced frequency are calculated according to equations (54) and (55), and their comparison with the master curves is shown in Figure 19. For the four asphalt mixtures, the storage modulus and the master curve almost overlap, but the loss modulus and of the master curve obtained by experiment will have a certain deviation at higher or lower frequencies. And all coefficients of determination R^2 are greater than 0.94. The storage modulus or loss modulus calculated by discrete and continuous relaxation spectrum is almost the same as R^2 . This shows that the discrete and continuous relaxation spectrum models can not only construct the relaxation modulus master curve with high accuracy, but also the obtained relaxation modulus master curve has little difference in accuracy.

4.5.1 Accuracy verification of the master curves of $J(t)$

According to Eq.(20) $E^*(\omega)$ and $J^*(\omega)$ relationship, it is deduced that the storage compliance $J'(\omega)$ and loss compliance $J''(\omega)$ in frequency are related to $E'(\omega)$ and $E''(\omega)$ are as follows:

$$J'(\omega)=\frac{E'(\omega)}{\left[E'(\omega)\right]^2+\left[E''(\omega)\right]^2} \tag{56}$$

$$J''(\omega)=\frac{E'(\omega)}{\left[E'(\omega)\right]^2+\left[E''(\omega)\right]^2} \tag{57}$$

$J'(\omega)$ and $J''(\omega)$ at reduced frequency are calculated using equations (56) and (57). The master curves of $J'(\omega)$ and $J''(\omega)$ are constructed again using the GSM and the approximate K-K relationship.

$J'(\omega)$ and $J''(\omega)$ for each reduced frequency are calculated using the relationship of the $J'(\omega)$ and $J''(\omega)$ of equations (58) and (59) to $L(\tau)$.

$$J'(\omega)=J_g+\int_{-\infty}^{+\infty}L(\tau)\frac{1}{\omega^2\tau^2+1}\mathrm{d}\ln\tau \tag{58}$$

$$J''(\omega)=\int_{-\infty}^{+\infty}L(\tau)\frac{\omega\tau}{\omega^2\tau^2+1}\mathrm{d}\ln\tau \tag{59}$$

The Eq. (58) and (59) are discretized by the chained trapezoidal rule, the integral interval is selected as $\left[10^{-40},10^{30}\right]$, and the subinterval length is selected as $\log\Delta\rho = 0.005$. The numerical model expressions of $J'(\omega)$ and $J''(\omega)$ with $L(\tau)$ are respectively:

$$J'(\omega) \approx \frac{1}{10^{\delta+\alpha}} + (\ln 10)\cdot\frac{0.005}{2}\cdot \left\{ \begin{array}{l} \dfrac{L(10^{-40})}{1+\omega^2(10^{-40})^2} \\ +2\left[\dfrac{L(10^{-39.995})}{1+\omega^2(10^{-39.995})^2}+\cdots+\dfrac{L(10^{29.995})}{1+\omega^2(10^{29.995})^2}\right] \\ +\dfrac{L(10^{30})}{1+\omega^2(10^{30})^2} \end{array} \right\} \tag{60}$$

$$J''(\omega) \approx \frac{1}{10^{\delta+\alpha}} + (\ln 10)\cdot\frac{0.005}{2}\cdot \left\{ \begin{array}{l} L(10^{-40})\dfrac{\omega 10^{-40}}{1+\omega^2(10^{-40})^2} \\ +2\left[L(10^{-39.995})\dfrac{\omega 10^{-39.995}}{1+\omega^2(10^{-39.995})^2}+\cdots+L(10^{29.995})\dfrac{\omega 10^{29.995}}{1+\omega^2(10^{29.995})^2}\right] \\ +L(10^{30})\dfrac{\omega 10^{30}}{1+\omega^2(10^{30})^2} \end{array} \right\} \tag{61}$$

$J'(\omega)$ and $J''(\omega)$ at each reduced frequency are calculated from the continuous retardation spectrum according to Eq. (60) and (61). However, $J'(\omega)$ and $J''(\omega)$ at each reduced frequency calculated from the discrete retardation spectrum can be directly obtained by using equations (23) and (24). Then plot the calculated $J'(\omega)$ and $J''(\omega)$ on a graph with master curves, as shown in Figure20. The master curves of $J'(\omega)$ and $J''(\omega)$ are almost overlapped, and the coefficients of determination R^2 are all greater than 0.96. And, $J'(\omega)$ and $J''(\omega)$ obtained from the discrete and continuous retardation spectrum are almost the same as the R^2. This shows that the transformation of relaxation spectrum to retardation spectrum in the time domain can obtain a high-precision creep modulus master curves. The creep compliance master curves constructed by discrete and continuous retardation spectrum has very little difference in accuracy.

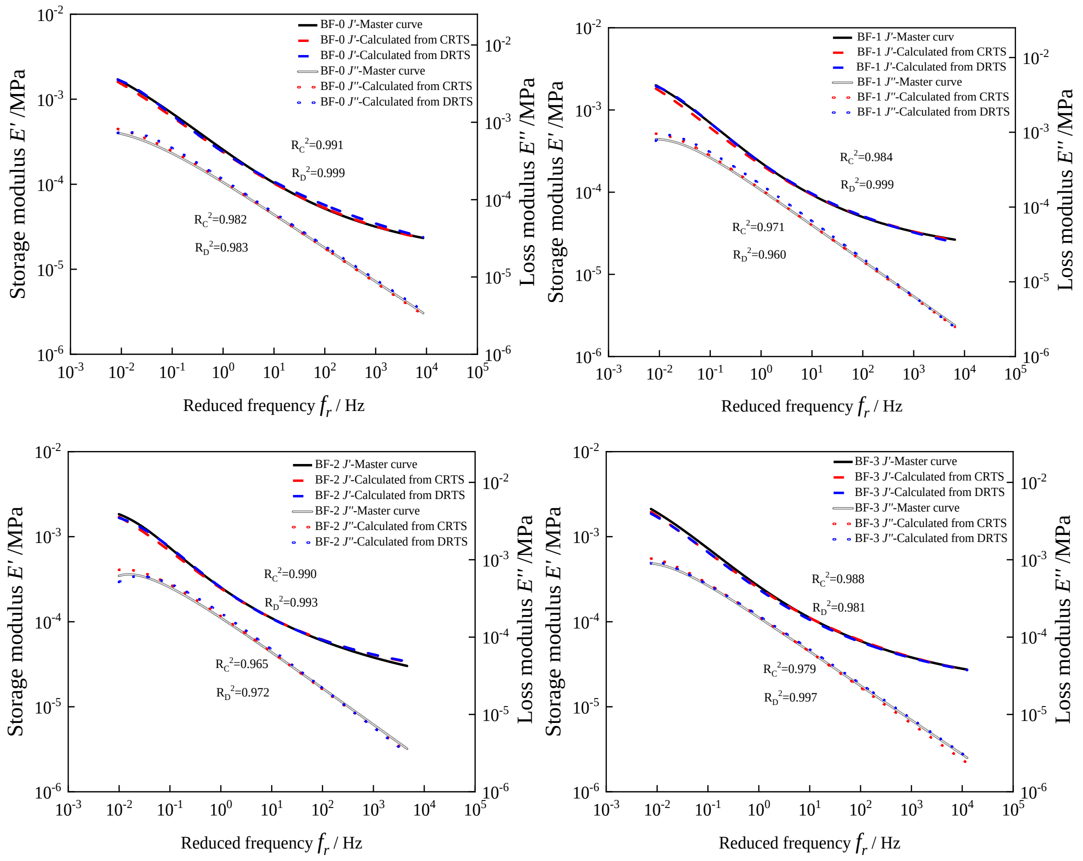

Fig.20. Comparison of calculated J' and J'' with the master curves of $J'(\omega)$ and $J''(\omega)$

5 Conclusion

In this paper, seven master curves of viscoelastic parameters are used to comprehensively characterize the viscoelastic behavior of basalt fiber asphalt mixture. The models of relaxation modulus and retardation compliance were determined using discrete and continuous spectrum models. The accuracy of the master curve of $E(t)$ and $J(t)$ obtained by the transformation was analyzed. The following conclusions are drawn:

(1) Basalt fiber can improve the high and low temperature performance of the asphalt mixture. The improvement of its low-temperature performance is mainly by strengthening the internal structure of the asphalt mixture.

(2) Discrete and continuous relaxation spectrum containing all the viscoelastic information were obtained from the master curves of $E'(\omega)$ and $E''(\omega)$ through the Prony series model and Laplace transform.

(3) The relaxation modulus and creep compliance master curve models can be easily obtained from the continuous relaxation and retardation spectrum models by using the the chained trapezoidal rule.

(4) Using both discrete and continuous models, the master curves of relaxation modulus and creep compliance can be obtained with high accuracy, and the obtained master curves have very little difference in accuracy.

(5) The asphalt mixture with the basalt fibers content of 0.2% has the best stress relaxation ability at low-temperature, and at the same time, has better deformation resistance at high-temperature.

Author Contributions: Huang Qi: Conceptualization, Investigation, Writing - Review & Editing.Xiaogang Kang: Formal analysis, Writing - Original Draft. Zhengji Zhang: Investigation. Chen Pengfei: Reviewed, and edited the manuscript. Zhuohang Zang：Conceptualization, Project administration. Erhu Yan: Resources.

Conflicts of Interest:

The authors declare no conflict of interest.

Data Availability Statement:

All data, models, and code generated or used during the study appear in the submitted article.

References

[1] 1 H. Booij, G.J.R.A. Thoone, Generalization of Kramers-Kronig transforms and some approximations of relations between viscoelastic quantities, 21(1) (1982) 15-24.

[2] 2 F. Zhang, L. Wang, C. Li, Y. Xing, The discrete and continuous retardation and relaxation spectrum method for viscoelastic characterization of warm mix crumb rubber-modified asphalt mixtures, Materials 13(17) (2020) 3723.

[3] 3 Biligiri, P. Krishna, Venudharan, Veena, Kaloush, E. Kamil, Chandrappa, K. Anush, Predictive Models for Storage Modulus and Loss Modulus of Asphalt Mixtures, Journal of Materials in Civil Engineering (2016).

[4] 4 H.A. Kassem, G.R. Chehab, S.S. Najjar, Quantification of the inherent uncertainty in the relaxation modulus and creep compliance of asphalt mixes, Mechanics of time-dependent materials 22(3) (2018) 331-350.

[5] 5 Q. Li, D.X. Xiao, K.C. Wang, K.D. Hall, Y. Qiu, Mechanistic-empirical pavement design guide (MEPDG): a bird's-eye view, Journal of Modern Transportation 19(2) (2011) 114-133.

[6] 6 K.H. Moon, M. Marasteanu, M. Turos, Comparison of thermal stresses calculated from asphalt binder and asphalt mixture creep tests, Journal of Materials in Civil Engineering 25(8) (2013) 1059-1067.

[7] 7 R.A. Schapery, A simple collocation method for fitting viscoelastic models to experimental data, (1962).

[8] 8 W. Zhang, B. Cui, X. Gu, Q. Dong, Comparison of relaxation modulus converted from frequency-and time-dependent viscoelastic functions through numerical methods, Applied Sciences 8(12) (2018) 2447.

[9] 9 W. Stark, The Phenomenological theory of linear viscoelastic behavior. An introduction. Von Nicholas W. Tschoegl. ISBN 3- 540- 19173- 9. Berlin/Heidelbere/New York/London/Paris/Tokyo/Hong Kong: Springer- Verlag 1989. XXV, 769 S., geb., DM 148, 00, Acta Polymerica 41(7) (1990) 425-425.

[10] 10 S. Park, R.J.I.j.o.s. Schapery, structures, Methods of interconversion between linear viscoelastic material functions. Part I—A numerical method based on Prony series, 36(11) (1999) 1653-1675.

[11] 11 M. Baumgaertel, H. Winter, Determination of discrete relaxation and retardation time spectra from dynamic mechanical data, Rheologica Acta 28(6) (1989) 511-519.

[12] 12 Y. Zhao, Y. Ni, W. Zeng, A consistent approach for characterising asphalt concrete based on generalised Maxwell or Kelvin model, Road Materials and Pavement Design 15(3) (2014) 674-690.

[13] 13 H. Liu, R. Luo, H. Lv, Establishing continuous relaxation spectrum based on complex modulus tests to construct relaxation modulus master curves in compliance with linear viscoelastic theory, Construction and Building Materials 165 (2018) 372-384.

[14] 14 A. Daoudi, D. Perraton, A. Dony, A. Carter, From complex modulus E* to creep compliance D (t): Experimental and modeling study, Materials 13(8) (2020) 1945.

[15] 15 Y. Cheng, D. Yu, Y. Gong, C. Zhu, J. Tao, W. Wang, Laboratory evaluation on performance of eco-friendly basalt fiber and diatomite compound modified asphalt mixture, Materials 11(12) (2018) 2400.

[16] 16 G. Tan, W. Wang, Y. Cheng, Y. Wang, Z. Zhu, Establishment of Complex Modulus Master Curves Based on Generalized Sigmoidal Model for Freeze–Thaw Resistance Evaluation of Basalt Fiber-Modified Asphalt Mixtures, Polymers 12(8) (2020) 1698.
[17] 17 A.T. 79-09, Standard Method of Test for Determining the Dynamic Modulus and Flow Number for Hot Mix Asphalt (HMA) Using the Asphalt Mixture Performance Tester (AMPT), AASHTO Washington, DC, 2011.
[18] 18 M. Jaczewski, J. Judycki, P. Jaskula, Asphalt concrete subjected to long-time loading at low temperatures–Deviations from the time-temperature superposition principle, Construction and Building Materials 202 (2019) 426-439.
[19] 19 M.L. Williams, R.F. Landel, J.D.J.J.o.t.A.C.s. Ferry, The temperature dependence of relaxation mechanisms in amorphous polymers and other glass-forming liquids, 77(14) (1955) 3701-3707.
[20] 20 G. Rowe, G. Baumgardner, M. Sharrock, Functional forms for master curve analysis of bituminous materials, Advanced testing and characterization of bituminous materials, two volume set, CRC Press2009, pp. 97-108.
[21] 21 D. Yu, X. Yu, Y. Gu, Establishment of linkages between empirical and mechanical models for asphalt mixtures through relaxation spectra determination, Construction and Building Materials 242 (2020) 118095.
[22] 22 Y. Zhu, L. Sun, H. Xu, L-curve based Tikhonov's regularization method for determining relaxation modulus from creep test, Journal of applied mechanics 78(3) (2011).
[23] 23 R. Anderssen, A.R. Davies, F. De Hoog, On the Volterra integral equation relating creep and relaxation, Inverse Problems 24(3) (2008) 035009.
[24] 24 N.W. Tschoegl, The phenomenological theory of linear viscoelastic behavior: an introduction, Springer Science & Business Media2012.
[25] 25 R. Schapery, S. Park, Methods of interconversion between linear viscoelastic material functions. Part II—an approximate analytical method, International Journal of Solids and Structures 36(11) (1999) 1677-1699.
[26] 26 S. Katicha, Analysis of hot mix asphalt (HMA) linear viscoelastic and bimodular properties using uniaxial compression and indirect tension (IDT) tests, Virginia Tech, 2007.
[27] 27 Y. Sun, J. Chen, B. Huang, Characterization of asphalt concrete linear viscoelastic behavior utilizing Havriliak–Negami complex modulus model, Construction and Building Materials 99 (2015) 226-234.
[28] 28 H. Liu, W. Zeiada, G.G. Al-Khateeb, A. Shanableh, M. Samarai, A framework for linear viscoelastic characterization of asphalt mixtures, Materials and Structures 53(2) (2020) 1-15.
