# Supplementary material for: Characterization of viscoelastic behavior of basalt fiber asphalt mixtures based on discrete and continuous spectrum models
Source: PLoS One. 2024 Feb 1;19(2):e0296087. doi: 10.1371/journal.pone.0296087 (PMC10833572; doi:10.1371/journal.pone.0296087)
Supplement: Attachment — Submitted filename: Reply.docx [file pone.0296087.s002.docx]

Dear Reviewers,

Thank you very much for your time involved in reviewing the manuscript and your very encouraging comments on the merits.

Comments: “The topic follows an interesting subject and appreciated work is done. The paper is well written.”

We also appreciate your clear and detailed feedback and hope that the explanation has fully addressed all of your concerns. In the remainder of this letter, we discuss each of your comments individually along with our corresponding responses.

To facilitate this discussion, we first retype your comments in italic font and then present our responses to the comments.

Reply to Reviewer #1

Comment 1:

*In the introduction, the paper has skipped explaining a brief background about the subject of the study and relevant literature in the field. Provide a clear background in the first one or two paragraphs.*

Response 1:

Thank you for your valuable comments, according to your comments, I have made changes in the introduction, the first paragraph clearly explains the road surface often encountered diseases, and the basalt fiber blending on the asphalt mixture structure improvement, and pointed out that the existing research on the viscoelastic behavior of the problem, more use of dynamic modulus and phase angle analysis of its viscoelastic behavior, rarely a comprehensive study of its viscoelastic properties. The second paragraph points out the conversion problem of the viscoelastic parameters of the mixture, and the third paragraph introduces the role of the time spectrum, and points out the disadvantages of the spectral discretization and the continuous time spectrum to determine the conversion of the main curve of viscoelastic parameters.

Comment 2:

*“Moreover, many studies characterizing the viscoelasticity…” You have not mentioned any study. Generally, the paper should clearly mention the shortcomings of the past literature to explain its own novelty better.*

Response 2:

In response to your comments, we have added some references in the first paragraph of the introduction that point out problems in existing research.

Comment 3:

*More explanation is needed on why the generalized sigmoidal model and approximate* *Kramers-Kronig relationship were selected.*

Response 3:

Thank you for your comment, I explained in lines 65-71 that the approximate Kramers-Kronig (K-K) relationship can model the dynamic modulus and phase angle master curves, the storage modulus and loss modulus master curves, and can represent the interrelationships of the viscoelastic parameters well, and that it is a simple and convenient way to represent the loss modulus master curves by using the approximate K-K relationship between the storage modulus and loss modulus process. The asphalt mixture master curves established by the Sigmoidal Model (GSM), which is capable of quasi-taking the dynamic mechanical response with high correlation coefficients and accurately predicting the dynamic mechanical properties, are clearly explained in lines 71-82. Simultaneous application of the approximate K-K relation and the GSM allows the construction of master curves that both ensure that the plotted master curve conforms to the line viscoelasticity and allow the master curve not to be symmetric about the viewpoint.

Comment 4:

*I suggest the theoretical background can be shortened. In the material and methods section, you can narrowly focus on the methodology and the research process. So, you can move parts of the formulations to the appendix or just mention them.*

Response 4:

Thank you for your comments, the background of this article accordingly made deletions, the article only retained the important formulas, the derivation process of the formula by the formula to text, so that the reader can make a clearer understanding of the process. In addition to this, in order to make the paper more readable and professional and to bring out the theme of viscoelasticity and fibers. Therefore, we have cut down the formulas that unnecessarily show the derivation process and made necessary adjustments to the syntax of the article. These adjustments do not affect the logic and science of the original paper.

Comment 5:

*Insert space before reference bracket.*

Response 5:

Thank you for your detailed review. We have carefully revised and reviewed this article for such problems as you mentioned.

Comment 6:

*Overall, the writing is clear, but carefully proofreading to fix minor typos and formatting issues would further polish the manuscript. Some sentences are long and could be broken up or restructured for clarity.*

Response 6:

Thank you for your comments, we have carefully and thoroughly proofread the manuscript, corrected all grammar and typos, and split longer sentences.

Reply to Reviewer #2

Comment 1:

*need to specify the problem statement clearly.*

Response 1:

Thank you for your valuable comments, according to your comments I have revised and adjusted the background in the introduction, now the introduction can be more clear description of the research background of this paper as well as the focus of the research.

Comment 2:

*Need to identify which layer(surface or binder or base) , the author recommend to use this type of fiber or in general.*

Response 2:

Line 95 describes that basalt fiber asphalt mixtures are commonly used in the upper and middle layers of asphalt pavements; lines 98-99 describe the type of material and source of basalt fibers, "Basalt fibers are selected from 9mm short-cut basalt fibers produced by Changsha North American Float Company."

Comment 3:

*need to clarify the utilized aspect ration of fiber.*

Response 3:

Line 100 of the article states that basalt fibers have an L/D ratio of about 560.

Reply to Reviewer #3

Comment 1:

*The comments are shown in the attached file.*

Response 1:

Thank you for your detailed comments, this paper has been carefully and meticulously revised according to the labeling information you attached to the errors in the article, such as the references of tables and figures in the text, as well as the description of the graphs and charts. Among them, for Fig. 2 and Fig. 3 marked in your article, it is further explained to you that the English terminology to express the frequency reduction is not a problem, and these two diagrams represent the phase angle and dynamic modulus of basalt fibers with different dopant amounts at different shrinkage frequencies, respectively, and then plotted as a master curve, so there is no error in the title and the transverse coordinates.

Comment 2:

*It is recommended to check some highlighted phrases in the results and check the number and citation of tables.*

Response 2:

Thank you for your detailed comments, I have changed the incorrect information in the conclusion labeling and have double checked and corrected the citations of the charts and added citations where they were missing and they are now all cited correctly and clearly.

Comment 3:

*In addition, it is highly recommended to cite more recent references.*

Response 3:

Based on your comments, I have included references to the latest articles.

We would like to take this opportunity to thank you for all your time involved and this great opportunity for us to improve the manuscript. We hope you will find this revised version satisfactory.

Sincerely,

Er-hu Yan
